# Impaired reach-to-grasp kinematics in parkinsonian patients relates to dopamine-dependent, subthalamic beta bursts

**DOI:** 10.1038/s41531-021-00187-6

**Published:** 2021-06-29

**Authors:** Matteo Vissani, Chiara Palmisano, Jens Volkmann, Gianni Pezzoli, Silvestro Micera, Ioannis U. Isaias, Alberto Mazzoni

**Affiliations:** 1 The BioRobotics Institute, Scuola Superiore Sant’Anna, Pisa, Italy; 2 Department of Excellence in Robotics and AI, Scuola Superiore Sant’Anna, Pisa, Italy; 3 Department of Neurology, University Hospital and Julius Maximilian University, Würzburg, Germany; 4 Centro Parkinson ASST G. Pini-CTO, Milan, Italy; 5 Bertarelli Foundation Chair in Translational Neuroengineering, Center for Neuroprosthetics and Institute of Bioengineering, Ecole Polytechnique Federale de Lausanne, Lausanne, Switzerland

**Keywords:** Parkinson's disease, Basal ganglia

## Abstract

Excessive beta-band oscillations in the subthalamic nucleus are key neural features of Parkinson’s disease. Yet the distinctive contributions of beta low and high bands, their dependency on striatal dopamine, and their correlates with movement kinematics are unclear. Here, we show that the movement phases of the reach-to-grasp motor task are coded by the subthalamic bursting activity in a maximally-informative beta high range. A strong, three-fold correlation linked beta high range bursts, imbalanced inter-hemispheric striatal dopaminergic tone, and impaired inter-joint movement coordination. These results provide new insight into the neural correlates of motor control in parkinsonian patients, paving the way for more informative use of beta-band features for adaptive deep brain stimulation devices.

## Introduction

The human hand is an extremely powerful tool and its remarkable skill is a main source of our evolutionary success. The related brain circuitry has been well studied, but our understanding is thus far limited mostly to cortical areas and stems from non-invasive imaging and recordings (i.e., functional MRI and PET or EEG/MEG studies), which either lack the spatial resolution for deep brain nuclei or the temporal resolution needed for investigating the neural coding underlying the different phases of the reach-to-grasp task (i.e., reaching, grasping, and pulling)^1–3^. Our knowledge regarding the contribution of the basal ganglia to human prehension is currently poor, although extensive clinical evidence indicates direct involvement of these brain structures. In particular, Parkinson’s disease (PD)—a neurological disorder predominantly characterized by striatal dopamine loss—leads to problems in timing, sequencing (e.g., hand pre-shaping), and planning of the reach-to-grasp motor program^4^ that are only partly resolved by dopamine replacement therapy^5–8^. High-frequency stimulation of the subthalamic nucleus (STN) also improves distinct aspects of the reach-to-grasp task, in particular the maximum velocity and execution time during the reaching period^9^ and the grip formation^9,10^. A recent study by Pötter-Nerger and colleagues^11^ also showed that subthalamic neurons of parkinsonian patients exhibit an increased firing rate during the reaching phase of self-paced, reach-to-grasp movements with respect to the resting condition, which correlated with movement velocity. The STN indeed plays a cornerstone role in the pathophysiology of PD. Excessive beta power (13–30 Hz) in the local field potential (LFP) recorded in the STN has been consistently described in parkinsonian patients, and the amplitude of such activity has been linked to motor impairment^12^. More recently, assessment of STN activity in PD has been refined by measuring the relative distribution of bursts in terms of duration and amplitude, with pathological beta activity consisting of longer duration, phasic bursts^13^. Such bursts are thought to restrict the capacity of the basal ganglia system to encode physiologically relevant information about intended motor actions^14,15^. In this context, one unresolved aspect remains the relationship between striatal dopamine deficiency, beta power, and beta-burst modulation and PD-related dysfunction in coordinated movements such as prehension. Accordingly, we have investigated the information carried by the STN in coding different kinematic aspects of the reach-to-grasp task in parkinsonian patients chronically implanted for deep brain stimulation (DBS), and their correlation with an imaging marker of the striatal dopaminergic denervation.

## Results

### Clinical presentation and molecular imaging findings

In the eight patients involved in this study (Supplementary Table 1), the clinical improvement due to dopaminergic medication and bilateral STN DBS was similar (67.5 ± 18.8 and 68.2 ± 11.0% on the Unified Parkinson’s Disease Rating Scale (UPDRS)-III scale, respectively; note that all data is reported as average ± standard deviation), supporting the diagnosis of idiopathic PD and the correct placement of the electrodes for DBS. As expected^16^, all patients showed a reduced dopamine reuptake transporter (DAT) binding value in the striatum (Supplementary Table 2). The level of inter-hemispheric striatal dopamine loss (asymmetry index [AI], see methods) was 30.5 ± 16.1% for the PD cohort and 2.6 ± 2.1% for a reference dataset of healthy subjects, thus indicating significantly stronger asymmetry in PD (Mann–Whitney *U*-test, *p* < 0.001).

### Spatiotemporal and coordinative aspects of the reach-to-grasp task

**Fig. 1:**
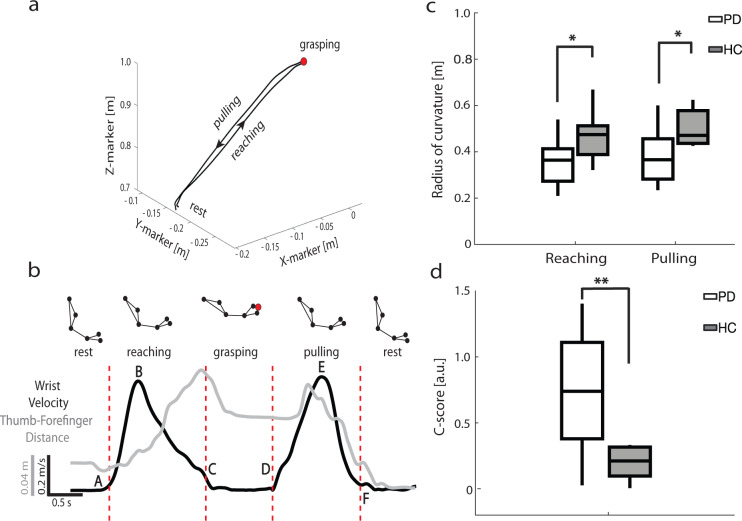
Kinematics measurements of parkinsonian patients (PD) and healthy controls (HC). **a** Example of the trajectory of one reach-to-grasp trial for patient wue02. The black arrows indicate the direction of the movement from the start of the reaching phase to the end of the pulling phase. X-marker: anterior–posterior coordinate of the marker; Y-marker: medio-lateral coordinate of the marker; Z-marker: vertical coordinate of the marker. **b** Task phases identification for patient wue02. Reach-to-grasp phases were defined according to the wrist marker absolute velocity profile (black line). The thumb-forefinger distance is plotted in gray. The letters A–F indicate the relevant timestamps during the reach-to-grasp task: A, movement onset; B, velocity peak of the reaching phase; C, approach to target; D, movement onset; E, velocity peak of the pulling phase; F, comeback to start position. The red dashed vertical lines identify the phases. Markers were placed on the acromion, the bicep muscle, the lateral epicondyle of the humerus, the ulnar styloid, the distal phalange dorsal head of the index, and the tip of the thumb. See also Supplementary Fig. 1. **c** Radius of the curvature during the reaching and pulling phases. **d** Coordination C-score. Boxplots are drawn between the 25th and the 75th percentiles, with a horizontal line indicating the median. The whiskers extended above and below to the most extreme data points within 1.5 times the interquartile range. Asterisks denote the statistical significance *p < 0.05, **p < 0.01, and ***p < 0.001 of the Mann–Whitney U-test. See also Supplementary Figs. 1–4.

All patients performed the reach-to-grasp protocol in the medication off and stimulation off condition (see “Methods” section). The same task was performed by a group of ten age-matched healthy controls (HC). Based on the acquired kinematic data, we divided the task in four phases: rest, reaching, grasping, and pulling (Fig. 1). Detailed kinematic measurements are reported in Supplementary Materials (Supplementary Figs. 1–4 and Supplementary Results). Of most interest, the radius of the curvature of the trajectory (measuring its smoothness, see “Methods” section) was lower in PD patients than HC both during reaching phase (PD 0.36 ± 0.04 m *n* = 8; HC 0.47 ± 0.03 m *n* = 10; Mann–Whitney *U*-test, *p* < 0.05) and pulling phase (PD 0.38 ± 0.04 m *n* = 8; HC 0.5 ± 0.03 m *n* = 10; Mann–Whitney *U*-test, *p* < 0.05; Fig. 1c). The radius of the curvature strongly correlated with the velocity profile in both groups (Supplementary Fig. 2a–c). The curvature–velocity correlation did not significantly differ between the two cohorts (PD  1.17 ± 0.15  *n* = 8; HC  1.13 ± 0.13 *n* = 10; Mann–Whitney *U*-test, *p* = 0.87; Supplementary Fig. 2d). The *C*-score (see Supplementary Fig. 4 and see “Methods” section) differed significantly between PD patients and HC (PD 0.73 ± 0.17 *n* = 8; HC 0.24 ± 0.04 *n* = 10; Mann–Whitney *U*-test, *p* < 0.01; Fig. 1d). This index, which specifically addresses the coordination between shoulder and elbow during the whole reach-to-grasp task, varied widely across patients from 0.026 (indicating smooth movement^17^) up to 1.404 (associated with a severe lack of coordination^17^).

### Low and high beta-frequency power modulation carries independent information about reach-to-grasp phases

**Fig. 2:**
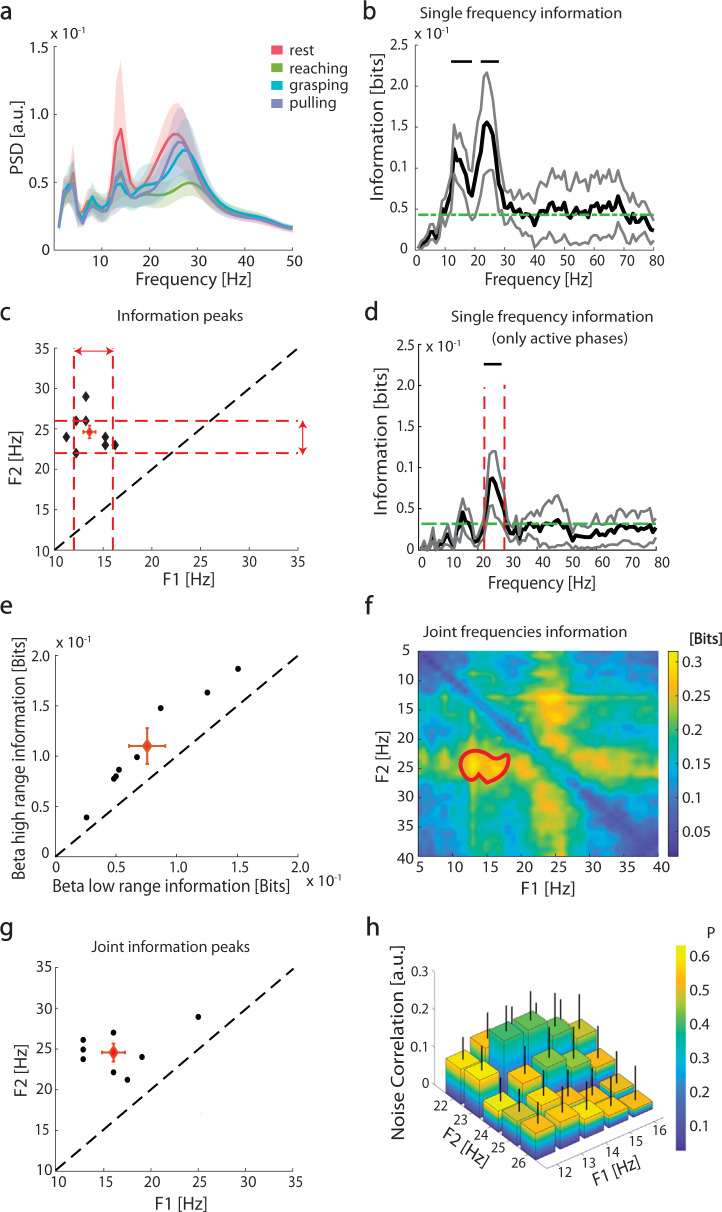
Subthalamic nucleus local field potential (LFP) spectral information about reach-to-grasp phases. **a** Group average subthalamic power spectral density (PSD) during the reach-to-grasp task. The shaded regions represent the standard errors of the means (SEM). **b** Group average frequency-wise spectral information of task phases carried by LFP power (black line). The gray lines represent SEM. The green dashed line is the significance threshold. Black horizontal upper lines denote the range of frequencies with a significant amount of information carried by the LFP power according the permutation test (PT). (*p* < 0.05, false discovery rate (FDR) correction). **c** Frequency location of the two peaks in spectral information for each subject (black diamonds). The red diamond is the centroid of the 2D distribution, with red bars representing the standard error of the centroid along the two dimensions. Red dashed lines indicate the ranges selected for the subsequent analyses: beta low range (horizontal red double-sided arrow, 14 ± 2 Hz) and beta high range (vertical red double-sided arrow, 24 ± 2 Hz). Black dashed line indicates identity. **d** Same as **b**, but with mutual information between single frequency power and the set of active movement phases (reaching grasping pulling), not including rest. Inset reports mutual information between this set of movement phases and the overall high beta power and low beta power. Only the former information is significant (*p* < 0.05, bootstrap test). Vertical lines indicate high beta range boundaries as defined in **c**. **e** Information carried by beta low range (14 ± 2 Hz) and beta high range (24 ± 2 Hz) about task phases. The red diamond is the centroid of the 2D distribution, with red bars representing the standard error of the centroid along the two dimensions. Black dashed line indicates identity. **f** Group average of the joint spectral information about task phases. The region enclosed in the red thick curve represents the 2D interval (12–18 and 22–26 Hz) of the frequencies space with a significant amount of information (cluster-based PT, *p < 0.05). **g** Pairs of frequencies with maximal joint information at the single-subject level. The red diamond is the centroid of the 2D distribution, with red bars representing the standard error of the centroid along the two dimensions. Black dashed line indicates identity. **h** Noise correlation between pairs of frequencies for the two ranges. Color represents associated *p*-values and error-bar SEM across subjects. See also Supplementary Figs. 5 and 6 and Supplementary Table 3.

We investigated how the temporal structure of STN activity encoded the task phases (rest, reaching, grasping, and pulling) by computing the spectral information of the LFP. The power spectrum was characterized by three main peaks: one at low frequencies (~4 Hz) and two in the beta band (~14 and ~25 Hz) (Fig. 2a). The low-frequency peak (i.e., ~4 Hz) did not carry phase-specific information, while the two peaks in the beta band were found to convey significant information about the task phases (bootstrap test *n* = 8, *p* < 0.05; Fig. 2b). The mutual information analysis further identified the two most informative frequencies, beta low (14.38 ± 0.702 Hz) and beta high (24.62 ± 0.8 Hz) —i.e., the frequencies at which the power changes carried the most information about the task phases (Fig. 2b, c and Supplementary Table 3). At a single patient level, the most informative frequencies differed from the peak frequencies, defined by the power spectral density (PSD). Information peak frequencies were more consistent and reliable than PSD peak frequencies across patients (Supplementary Fig. 5). We defined the two most informative ranges as the most informative frequency ± 2 Hz, i.e., the beta low range (14 ± 2 Hz) and beta high range (24 ± 2 Hz) (dotted red lines in Fig. 2c). We refer to “beta low range” and “beta high range” below to indicate the two frequency ranges defined by information measurements, whereas conventional frequency intervals are referred to as “bands”, i.e., beta low band (13–20 Hz), beta high band (21–30 Hz), and whole beta band (13–30 Hz). The beta high-range power was significantly modulated by the task phases, while the beta high-band power did not display significant modulation (Supplementary Fig. 6a, b).

In a second set of analyses, we computed the information only for the active phases (reaching, grasping, and pulling), excluding rest (see “Methods” section). Significant information about active phases was carried by the beta high range, but not the beta low range (Fig. 2d). The information carried by the beta high range linearly correlated across subjects with the power change (as percentage vs. rest, see “Methods” section) (*R*^2^ = 0.82, *p* = 0.001). Such correlation was not observed for beta low range (*R*^2^ = 0.23, *p* > 0.1). Coherently, the beta high range carried more information than the beta low range when all the task phases, including rest, were considered (0.0747 ± 0.0032 bits vs. 0.042 ± 0.005 bits, *n* = 8, respectively; PT, *p* < 0.01; Fig. 2e).

It is nevertheless worth noting that both frequency ranges (i.e., high beta range and low beta range) showed a distinctive and independent contribution towards coding the task phases (including rest) (Fig. 2f, g). Indeed, optimal information about the task phases could be retrieved by a combination of the beta high and beta low ranges (Fig. 2f) for a total joint information of 0.32 bits and a very low redundancy of 0.003 bits. This was confirmed at a single subject level, where the highest joint information was carried by a combination of the peak frequencies of the two ranges (the centroid across subject [red diamond in Fig. 2g] was at 16 ± 0.9 and 24.5 ± 1.4 Hz). The frequencies in the two ranges shared a very low fraction of common noise-driven variance: the noise correlation between was always lower than *R* = 0.177 (Fig. 2h). Overall, these analyses revealed that selected frequency ranges within beta low and beta high bands are largely independent channels of information in the encoding of the task phases, with the beta high range mainly conveying information regarding active movements phases.

### Amplitude and duration modulation of beta bursts are informative of the task phases

**Fig. 3:**
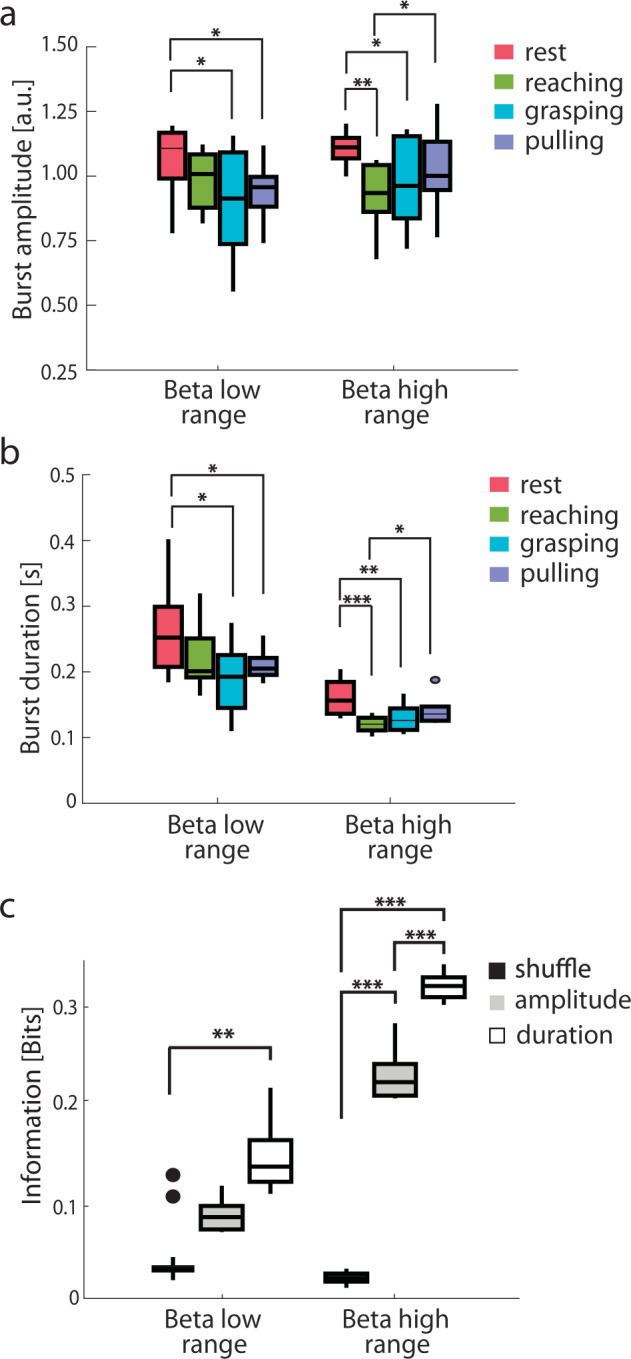
Burst analysis for beta ranges selected according to information analysis. **a** Group median amplitude of the bursts in beta low range (14 ± 2 Hz) and beta high range (24 ± 2 Hz) during the reach-to-grasp task. **b** Same as **a** for burst duration. **c** Group median information about the reach-to-grasp task carried by the amplitude (gray) and the duration (white) of the beta bursts. Information carried by a shuffle permutation (black) served for statistical significance. Boxplots are drawn between the 25th and the 75th percentiles, with a horizontal line indicating the median. The whiskers extended above and below to the most extreme data points within 1.5 times the interquartile range. Asterisks denote the statistical significance *p < 0.05, **p < 0.01, and ***p < 0.001 of the permutation test (PT). See also Supplementary Figs. 7 and 8.

We then investigated whether the task-related information carried by the power of the beta band ranges could be encoded in a dynamic modulation of beta synchronization, as reflected by beta bursts. In the beta low range, the bursts amplitude was significantly higher at rest (1.063 ± 0.05 a.u.) than during grasping (0.89 ± 0.08 a.u.; PT, *p* = 0.03) or pulling (0.941 ± 0.04 a.u.; *n* = 8; PT, *p* = 0.04). In the beta high range, the bursts amplitude was significantly higher at rest (1.107 ± 0.02 a.u.) than when reaching (0.927 ± 0.05 a.u.; *n* = 8; PT, *p* < 0.001) or grasping (0.9756 ± 0.064 a.u.; *n* = 8; PT, *p* = 0.04). The bursts amplitude in the beta high range was also significantly different between the two active phases of the reach-to-grasp, i.e., reaching and pulling (1.025 ± 0.06 a.u.; *n* = 8; PT, *p* = 0.02) (Fig. 3a).

Bursts duration modulation paralleled the changes in bursts amplitude. In the beta low range, the bursts duration was significantly longer at rest (0.26 ± 0.02 s) than during grasping (0.19 ± 0.02 s; PT, *p* < 0.05) or pulling (0.21 ± 0.01 s.; *n* = 8; PT, *p* < 0.05). In the beta high range, the bursts duration was significantly longer at rest (0.16 ± 0.01 s) than when reaching (0.12 ± 0.01 s; *n* = 8; PT, *p* < 0.001) or grasping (0.13 ± 0.01 s; *n* = 8; PT, *p* < 0.01). It also differed significantly between reaching and pulling (0.14 ± 0.01 s; *n* = 8; PT, *p* < 0.05) (Fig. 3b). The bursts duration of the beta low range carried significant information about the task phases (*I* = 0.154 ± 0.021 bits; *n* = 8; PT, *p* = 0.01), as did the bursts amplitude (*I* = 0.23 ± 0.01 bits; *n* = 8; PT, *p* < 0.001) and duration (*I* = 0.33 ± 0.07 bits; *n* = 8; PT, *p* < 0.001) of the beta high range (Fig. 3c). In the beta high range, the burst duration was more informative than the bursts amplitude (*n* = 8; PT, *p* < 0.001) (Fig. 3c). The information carried by beta high-range bursts amplitude and duration correlated with their percentage change compared to rest (*R*^2^ = 0.62, *p* < 0.05; *R*^2^ = 0.62, *p* < 0.05, respectively). This was not true for beta low-range bursts amplitude and duration (*R*^2^ = 0.03, *p* > 0.50; *R*^2^ = 0.48, *p* = 0.06, respectively).

Bursts computed in the whole beta band and in the conventional low and high beta bands displayed a weaker modulation in amplitude associated with task phases (Supplementary Fig. 7a), but similar bursts duration modulation with respect to the beta ranges (Supplementary Fig. 7b). Overall, conventional bands carried significant information, but less than the beta high range and beta low range (compare Supplementary Fig. 7c to Fig. 3c). No modulation, and hence no information about task phases, was present in the theta (4–8 Hz) and low gamma (40–60 Hz) burst features (Supplementary Fig. 8).

These results suggest that bursts features, especially when studied in maximally informative frequency ranges, carry more information than power modulations in coding specific task phases of the reach-to-grasp movement.

### Beta high-range bursts reflect pathological kinematics

**Fig. 4:**
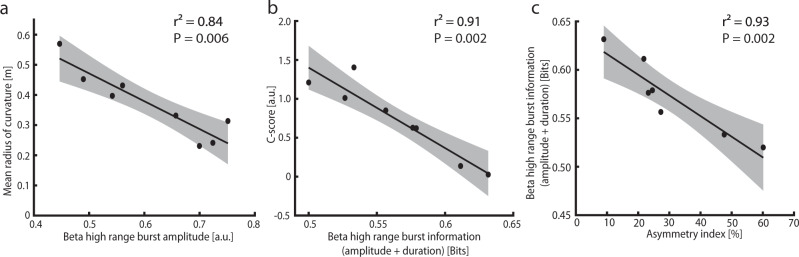
Correlation between kinematic aspects, burst information, and striatal dopamine reuptake transporter binding. **a** Correlation between the mean radius of the curvature and the amplitude of the bursts in the beta high range. **b** Correlation between the inter-joint coordination index (*C*-score) and the overall information (amplitude and duration) of the task phases carried by the bursts in the beta high range. **c** Correlation between the overall information (amplitude and duration) of the task phases carried by the bursts in the beta high range and the inter-hemispheric striatal dopamine loss (asymmetry index). In all panels, a linear regression (black line) is plotted with 95% confidence intervals (shaded gray regions). See also Supplementary Fig. 9.

Since only beta high-range bursts amplitude and duration were informative about the reach-to-grasp phases, these were further correlated with PD-specific kinematic alterations. We found a strong negative correlation between high beta range bursts amplitude and the mean radius of the curvature (*R*^2^ = 0.84; *n* = 8; *p* = 0.006; Fig. 4a); a larger amplitude of the bursts in the high beta range was associated with a more irregular trajectory of the movement. We did not find a significant correlation between high beta range burst amplitude and both the wrist velocity and time to velocity peak (see Supplementary Table 4a, b).

We also found a strong negative correlation between the information carried by the combined bursts amplitude and bursts duration of the beta high range and the *C*-score (*R*^2^ = 0.91; *n* = 8; *p* = 0.002; Fig. 4b); the subjects in which beta high range bursts carried more information where the ones with better movement coordination. Of note, the percentage modulation of beta high-range burst duration and amplitude with respect to rest did not significantly correlate with the *C*-score (*R*^2^ = 0.23, *p* = 0.22; *R*^2^ = 0.22, *p* = 0.25; *R*^2^ = 0.24, *p* = 0.22 for the two features independently or combined, respectively, Supplementary Table 5), suggesting that what is relevant is the amount of effective information conveyed by bursts modulation (which also considers the variability of the signal) and not the entity of the modulation.

### Imbalanced inter-hemispheric striatal dopamine tone relates to beta high range bursts

Finally, we investigated the correlation between striatal dopaminergic loss, subthalamic activity, and the resultant kinematic abnormalities.

We found a significant negative correlation between the DAT binding values of the contralateral striatum and the time to reach the peak velocity during the reaching phase (*R*^2^ = 0.7, *R* = −0.84; *n* = 7; *p* = 0.01). No other significant correlations were found between DAT binding values and kinematic features (Supplementary Table 6a, b). Striatal DAT binding values did not correlate with beta power and any burst features (Supplementary Table 7). Instead, The asymmetry index (AI) of striatal DAT binding values (see “Methods” section) strongly correlated with the *C*-score (*R*^2^ = 0.67; *n* = 7; *p* = 0.02; Supplementary Fig. 9). Coherently with these results, the AI was also associated with a strong and significant decrease of beta high range bursts information in coding the reach-to-grasp task phases (*R*^2^ = 0.93; *n* = 7; *p* = 0.002; Fig. 4c); the higher the inter-hemispheric asymmetry of striatal dopamine loss, the less effective the inter-phase bursts modulation (i.e., the lower its information content), and the worse the movement coordination.

## Discussion

We have shown that an imbalanced inter-hemispheric striatal dopamine loss in parkinsonian patients is associated with poor subthalamic beta high bursts coding of prehension, particularly in terms of movement coordination. We have also found that subthalamic beta bursts dynamics is more informative than power modulations in coding the reach-to-grasp task. Moreover, beta low and beta high oscillations carry independent information about the reach-to-grasp task, with beta high being informative of the active phases (i.e., reaching, grasping, and pulling).

Mutual information^18^ is a relevant measure for neural coding for two reasons. First, it quantifies the reduction of uncertainty about the stimulus that can be gained from the observation of a single trial of the neural response, rather than from the observation of the average values^19^. This reflects the ability to execute a movement based on online decoding of neural features. Second, mutual information is the optimal way to take inter-condition differences and inter-trial variability into account without any a priori assumptions about the distribution of the variables^19^ or the relationship between them. This makes mutual information particularly useful when trying to understand the complex dynamics of motor control such as movement coordination. These two properties enable mutual information to provide an upper bound to decoding algorithms operating on the same dataset^20^. Spectral information analysis can therefore help in the design of novel decoding algorithms^20^ for a richer palette of input signals^23,24^ in new DBS devices capable of real-time adaptation of the stimulation delivery (adaptive DBS)^21,22^.

Despite being one of the most essential motor behaviors of daily living, reach-to-grasp movements have been poorly investigated in PD, with more attention given to simple movements such as finger tapping^25^, pressing a button^26^, gripping^27^, or wrist extension then flexion^28^ and guided movements (using a joystick, a rotational handle, etc.)^29,30^. Different paradigms rather than self-paced movements have been also been preferred to study STN activity, such as the warned reaction time task^26,28,30^, directed or warning cueing^31^, time or velocity constraints^5^, or the concurrent spatial-visualization task^32^. Some studies were only performed while patients were on their usual medication state^27,30,33,34^, which cannot be assumed to be a normalized state—especially after a long-term pulsatile levodopa treatment^35^ and the direct effect of levodopa on extrastriatal motor areas^36,37^. Almost all previous reports used intraoperative recordings or LFP data collected from the DBS electrodes in the immediate postoperative period (within 10 days), which are influenced by high impedance variability and the microlesioning effect^27,38^. Our study differs from most of the previous reports, as it uses subthalamic LFP recordings months after surgery. Recordings were acquired from the chronically-active electrodes that were most effective in improving motor symptoms. Also, we aimed to study the reach-to-grasp task as usually performed in everyday life, and to provide new evidence of the role of the basal ganglia in prehensile actions by combining neurophysiological measurements and molecular imaging findings.

Our study has some limitations. In particular, the severity of symptoms limited the pausing of DBS to 2 h before the start of the study. Nevertheless, this time was sufficient for all patients to reach a motor impairment similar to the pre-DBS condition (Supplementary Table 1). To limit the discomfort of patients due to the long study protocol, we performed the task only with the dominant hand and only recorded the subthalamic activity contralaterally; therefore, our study did not account for the laterality of the symptoms.

In line with previous kinematic studies, our parkinsonian patients displayed bradykinetic motor behavior characterized by decreased peak velocity^4,6,31,39^ and longer time to peak velocity^4^ (Supplementary Fig. 3a, b). The latter measurement might particularly reflect the symptom bradykinesia, given the strong dependency from dopamine deficiency of the contralateral striatum (Supplementary Table 6a)^40–43^.

Impairment of the movement trajectory and poor pre-shape coordination have also been observed in PD^6,39,44^, and associated with a deficit in the implementation of sequential movements by parkinsonian patients^45,46^. Our patients also showed an increased C-score index (Fig. 2). This measurement was an adequate reflection of the individual coordination (Supplementary Fig. 3), and strongly correlated with neurophysiological and molecular imaging findings (Fig. 4 and Supplementary Fig. 9). Patients with PD do exhibit poor integration of subsequent movement phases (altered joint-based coordinate frame), possibly due to impaired egocentric representation as a consequence of the key role of the basal ganglia in the parietofrontal and dorsal premotor circuits involved in the processing of somatosensory transformation for prehension^1,33,47,48^.

Beta bursts represent a communication channel in the cortical-basal ganglia network encoding physiologically-relevant information about intended motor actions^14,15,49^. Notably, Feingold and colleague^15^ showed in healthy nonhuman primates that maximum beta bursting in the striatum occurs after reward and task end, thus allowing evaluation of the motor action performance (retrospective evaluation) in terms of expected rewards and anticipated energetic demands. Reduced striatal dopamine results in poor estimation of the desirability of action (energetic cost-benefit trade-off)^50^.

Bursts modulation in PD could also account for compensatory attempts at the STN level to preserve proper information coding for the execution of a desired motor task^15,51–54^. Reinterpreting subthalamic beta dynamics in PD as a compensatory mechanism could possibly explain the lack of a direct correlation with striatal DAT loss. Indeed, such a mechanism might be more susceptible to the dopamine deficiency threshold or, more likely, follow inter-hemispheric basal ganglia circuitry derangements as captured by the AI, especially for the coordinative aspects of motor tasks (Fig. 4 and Supplementary Fig. 9)^16,55,56^. In support, bilateral activation of the putamen has been described during reaching movements^57^.

In line, subthalamic beta power fluctuations parallel the improvement in bradykinesia and rigidity due to levodopa treatment^58,59^ and STN-DBS^60–62^ rather than their clinical severity, which is instead truly dopamine dependent^40–43^. Such fluctuations could indeed reflect the magnitude of the subthalamic compensatory attempt, then replaced by levodopa and STN-DBS. Similarly, when other compensatory mechanisms (and networks) are in place, such as the noradrenergic system in PD-tremor^63–65^, the subthalamic contribution would be downturned or overwritten and fewer beta oscillations recorded^62,66^. This reasoning could also explain poor beta oscillations in PD during other behaviors, such as sleep, despite the maintenance of severe bradykinesia and rigidity^67^. Indeed, when the STN is involved in the ascending activating network implicated in the transmission of the so-called PGO (ponto-geniculo-occipital) waves during REM sleep, enhancements of subthalamic beta oscillatory activity are associated with muscular atonia^68^.

Another relevant finding of our study is that beta low and beta high oscillations carry independent information about the reach-to-grasp task (Fig. 2), with beta low oscillations conveying information about the state (rest vs. movement) and beta high oscillations relating to the active phases (reaching, grasping, and pulling) of the reach-to-grasp task (Figs. 2g and 3d). The STN is a cornerstone of multiple re-entrant cortico-subcortical pathways, with a strong functional relationship with both the striatum and cortical areas^69^. The distinctive contribution of beta low and beta high bursts might suggest a frequency-dependent regional contribution of the cortico-basal ganglia object-grasping network^70^. The finding that beta high bursts predominantly code the coordinative aspects of the reaching phase would suggest preferential involvement of motor cortical areas in the high beta frequencies^71^. Low beta rhythms are instead the dynamic substrate for sensorimotor processing of parietal areas^71,72^. Given the relatively poor task constraints and perceptual discordances of the motor tasks applied in this study, a predominant contribution of frontal areas would be expected. Future studies with more complex reach-to-grasp paradigms and combined cortical and subthalamic recordings will possibly elucidate these assumptions.

Our findings are of particular value for new adaptive DBS devices^21,22^. In this context, adaptive systems that rely on the level of beta oscillations should modulate rather than suppress beta activity, so as not to impair the correct coding of volitional movements, as nicely shown by Iturrate and coll.^73^ Accordingly, devices that aim to modulate the duration or amplitude of beta bursts^74^ should preserve the (residual or compensatory) specific physiological information carried to code kinematic features of (arm) movements^75^.

## Methods

### Patients and surgery

All eight patients (Supplementary Table 1) enrolled in this study were diagnosed with idiopathic Parkinson’s disease (PD) according to the UK Parkinson Disease Brain Bank criteria^76^, and evaluated using the Unified Parkinson Disease Rating Scale motor part (UPDRS-III). The local Institutional Review Board of the University Hospital of Würzburg approved the study and all patients gave written informed consent according to the Declaration of Helsinki.

Patients were selected based on established criteria for deep brain stimulation (DBS) surgery^77^ and were implanted at the University Hospital of Würzburg with the Activa PC + S® neurostimulation system (Medtronic, PLC). This system allows therapeutic DBS as well as on-demand LFP recordings from the implanted subthalamic nucleus (STN) electrodes. At the time of the experiment (10 ± 1 [9–12] months after surgery), all patients were on stable dopaminergic treatment (for at least 2 months) and chronically stimulated (i.e., unchanged DBS parameters for at least 2 months). The surgical procedure has been previously described^78^. The intended coordinates for STN (i.e., 12 mm lateral, 2 mm posterior, and 4 mm ventral to the mid-commissural point) were adjusted according to individual delineation of the STN on T2-weighted and susceptibility-weighted MRI (Siemens MAGNETOM Trio 3.0 T). Intraoperative microelectrode recordings and stimulation and intraoperative CT scans confirmed the targeting. The precise localization of the active contacts used for chronic stimulation and LFP recordings was further confirmed by image fusion of pre-operative and postoperative scans (SureTune^TM^, Medtronic, PLC). Correct placement of the electrodes was also verified by the clinical response to DBS (medication off/stimulation on) compared to the preoperative improvement in the UPDRS-III score during levodopa challenge test (medication off vs. medication on). The therapeutic response to DBS or levodopa was expressed as the percentage of improvement, according to the formula ((*a* − *b*)/*a*) × 100 (adapted from ref. ^79^), where *a* is the medication-off UPDRS-III score and *b* is the medication-on UPDRS-III score pre-DBS or *b* is the medication-off/stimulation-on UPDRS-III score at the time of the test (post-DBS).

### Reach-to-grasp task

All participants started the tasks comfortably seated on a chair (the feet and the back were supported), with both hands resting on the table surface. The starting position of the arm and hand was with the shoulder in neutral position, the elbow flexed (~90°), the forearm in mid-pronation, and the ulnar border of the hand resting upon the table. The index finger and thumb were held in a relaxed position of opposition. Participants were instructed to reach towards and grasp a target with their dominant hand (the right hand for all subjects). The target was a small sphere of 12 mm diameter placed vertically (at the top of a metallic stick) opposite to the (right) midclavicular line, at the height of the acromion and at a distance from the patient equal to the distance from their acromion and ulnar styloid (with the arm stretched out). We asked patients to perform the movement as they would normally do at home to reach-to-grasp an object, and to remain in the rest position for some seconds between each reach-to-grasp movement (Fig. 1a). For each patient, we recorded three blocks of ten trials each. Subjects were allowed to rest after each block. All patients were tested in the morning, at least 12 h after their last dose of antiparkinsonian medication and 2 h after pausing the stimulation (i.e., medication off/stimulation off condition). A group of ten age-matched healthy controls (seven males, three females; age range 56–70 years) performed the same test.

### Kinematic analysis

Kinematics was measured with a motion capture system (SIMI Motion 3D, SIMI Reality Motion Systems GmbH or SMART-DX, BTS Bioengineering). Six retro-reflective markers were placed on the acromion, the bicep muscle, the lateral epicondyle of the humerus, the ulnar styloid, the distal phalange dorsal head of the index finger, and the tip of the thumb of the dominant (right) arm. The marker coordinates were low-pass filtered (cut-off frequency of 8 Hz) and smoothed using a Savitzy-Golay filter of 30-samples window. Coordinates tracks were numerically differentiated (i.e., forward first-order differentiation) to obtain marker velocities (Fig. 1b and Supplementary Fig. 1). Specific sets of parameters were then automatically extracted by Matlab^TM^-based custom scripts and checked by visual inspection. For each subject, variables were averaged over the trials. Based on the velocity of the marker placed on the wrist, for each reach-to-grasp trial we identified six relevant events (Fig. 1b) using a noise-adaptive threshold centered around 0.05 m/s. The four phases: rest, reaching, grasping, and pulling were identified based on these events (Fig. 1). We calculated the peak velocity of the wrist marker and the time to reach the peak velocity. To define the trajectory of the reach-to-grasp movement, we measured the radius of the curvature of each instant i as the radius *R*_i_ of the circle passing through the corners of the triangle formed by the neighboring points *P*_i−1_, *P*_i_, and *P*_i+1_, where each *P*_i_ = [*x*_i_*, y*_i_*, z*_i_] is given by the three dimensional coordinates of the trajectory at time i. *R*_i_ can be derived using the following equation:

1 $$\begin{array}{l}R_{\rm{i}} =\\ \left\| {\frac{{\left\| {P_{\rm{i}} - P_{{\rm{i}}\, + \,1}} \right\|^2(P_{{\rm{i}} - 1} - P_{\rm{i}}) \times (P_{{\rm{i}} + 1} - P_{\rm{i}}) \times (P_{{\rm{i}} - 1} - P_{\rm{i}}) - \left\| {P_{\rm{i}} - P_{{\rm{i}} - 1}} \right\|^2(P_{{\rm{i}} - 1} - P_{\rm{i}}) \times (P_{{\rm{i}} + 1} - P_{\rm{i}}) \times (P_{{\rm{i}} + 1} - P_{\rm{i}})}}{{2\left\| {(P_{{\rm{i}} - 1} - P_{\rm{i}}) \times (P_{{\rm{i}} + 1} - P_{\rm{i}})} \right\|^2}}} \right\|,\end{array}$$

where × represents the cross-product between two vectors and $\left\| \cdot \right\|$ is the norm of the vector (Supplementary Fig. 2). The curvature is the inverse of the radius of the curvature.

We then analyzed the coordinative aspects of the reach-to-grasp task. We measured for each trial the peak hand aperture:

2 $${\mathrm{PHA = }}\frac{{\max [d_{{\rm{i}} - {\rm{h}}}]_{{\rm{reach}}} - \min [d_{{\rm{i}} - {\rm{h}}}]_{{\rm{grasp}}}}}{{100 \ast \max [d_{{\rm{i}} - {\rm{h}}}]_{{\rm{reach}}}}},$$

where *d*_i−h_ is the distance between the index finger and the thumb and max[*d*_i−h_]_reach_ and min[*d*_i−h_]_grasp_ its maximal value during reach and minimal value during grasp, respectively. A value of peak hand aperture close to 100% indicates a large aperture during reach compared to the size of the object. We also calculated the pre-shape coordination index (PCI) as follows:

3 $${\mathrm{PCI = }}1 - \frac{{{{t}}_{\mathrm{p}} - {{B}}}}{{{\Delta} {{t}}_{{\mathrm{tot}}}}}$$

where *t*_p_ is the instant of the peak hand aperture, *B* is the instant of the peak velocity of the wrist marker, and Δ*t*_tot_ is the duration of the reaching and grasping phases together. This index represents a measure of how well one subject coordinates the hand and arm during the reach-to-grasp movement. Normally, subjects pre-shape their hand during reaching so that their hand configuration evolves gradually to conform to the size and shape of the object to be grasped^80^. In highly coordinated movements, the hand achieves peak aperture close to the instant of peak speed, reflecting an integration of these two components in mapping the motor action to the object. Larger values reflect increased separation between peak hand aperture and peak speed, and thus a poorer pre-shape coordination. Finally, we computed the coefficient *C*-score^17^. This represents the linear approximation of the synergies between the shoulder and elbow angular velocities. Low *C*-score values are correlated to higher kinematic synergies and vice-versa. Details of the computation of this index have been described elsewhere^17^. In brief, shoulder (*α*) and elbow (*β*) angles and their derivates were calculated from the coordinates of the acromion, the lateral epicondyle of the humerus and the ulnar styloid. Movements were represented in terms of a shoulder vs. elbow angular velocities plot, creating a two-lobed plot: one for the reaching and one for the pulling phase. We measured the *C*-score as the angular coefficient of the line connecting the position of the centroids of the two lobes (Supplementary Fig. 3).

### Molecular imaging study

Molecular imaging data acquisition, reconstruction, and analysis have been described previously^16,81^. All patients apart from one (wue05 refused the exam) underwent single-photon computed tomography (SPECT) with [^123^I]N-ω-fluoropropyl-2β-carbomethoxy-3β-(4-iodophenyl)nortropane (FP-CIT) to measure the striatal DAT density. SPECT studies were performed within 3 months before surgery. Based on the DAT availability, we computed an asymmetry index (AI) for striatal non-displaceable binding potential (BPND):

4 $${\mathrm{AI = }}\frac{{{\mathrm{BPND}}^{{\mathrm{IPSI}}} - {\mathrm{BPND}}^{{\mathrm{CONTRA}}}}}{{{\mathrm{BPND}}^{{\mathrm{IPSI}}} + {\mathrm{BPND}}^{{\mathrm{CONTRA}}}}} \times 200,$$

where contra (contralateral) refers to the side opposite to the clinically most impaired hemibody and ipsi (ipsilateral) to the other side. Striatal DAT binding measurements were compared with normative data of 15 healthy subjects (four males, 11 females; age range 44–68 years). For these subjects, we adopted the convention of referring to the right side as ipsilateral^82^.

### Electrophysiological signal recording and pre-processing

Subthalamic LFP were recorded from the STN contralateral to the dominant hand used to perform the task, using a single bipolar contact configuration and amplified by 1000. The recording contacts were chosen according to the chronic stimulation setting as a bipolar montage of the two contacts surrounding the stimulation cathode. Synchronicity across STN recordings and kinematic measures and artifacts management (including ECG artifact) were ensured as previously described^81,83^. LFP signals were acquired at sampling frequency of 422 Hz, then resampled at 250 Hz and bandpass filtered around 1–100 Hz using a zero phase-delay, 5th-order Butterworth filter. A notch filter at 50 Hz with a high-quality factor Q (i.e., *Q* = 50) was also applied. We then applied a *z*-score normalization on the LFP signal to allow inter-subject comparison, and to reduce variability induced by distances between electrodes and neural sources in different implants.

### Spectral and mutual information analysis of local field potential recordings

We analyzed the STN activity during the rest, reaching, grasping, and pulling phases by means of a time-frequency decomposition using 50 Morlet wavelets from 1 to 50 Hz (ten cycles). We then cut the dataset around each phase and averaged across time to obtain the marginal power spectral density. To assess how well the power of the LFP encodes for the reach-to-grasp task (i.e., rest, reaching, grasping, and pulling), we performed a mutual information analysis between the LFP power and each of two sets of behavioral phases PH: all task phases {rest, reaching, grasping, and pulling} and active phases only {reaching, grasping, pulling}. The spectral information *I*(PH; *R*_f_) quantified how much information the power of the LFP *R*_f_ for a given frequency *f* carried about the set of behavioral phases PH as follows:

5 $${\mathrm{I}}\left( {{\mathrm{PH}};{\mathrm{R}}_{\mathrm{f}}} \right) = \mathop {\sum}\nolimits_{{\mathrm{ph}}} {{\mathrm{P(ph)}}\mathop {\sum}\nolimits_{{\rm{r}}_{\rm{f}}} {{\mathrm{P}}\left( {{\mathrm{r}}_{\mathrm{f}}\left| {{\mathrm{ph}}} \right.} \right){\mathrm{log}}_2{\textstyle{{{\mathrm{P}}\left( {{\mathrm{r}}_{\mathrm{f}}|{\mathrm{ph}}} \right)} \over {{\mathrm{P}}\left( {{\mathrm{r}}_{\mathrm{f}}} \right)}}}} },$$

where *P*(ph) is the probability of the occurrence of the phase ph, *P*(r_f_) is the probability of the frequency *f* to have power *r*_f_ over all trials and all phases, and *P*(r_f_|ph) is the probability of the power *r*_f_ to occur during phase ph. The joint information about the reach-to-grasp phases PH carried by the combination of the power of the frequencies Rf1 and Rf2 was computed as follows:

6 $${\mathrm{I}}\left( {{\mathrm{PH}};{\mathrm{R}}_{{\mathrm{f}}1}{\mathrm{R}}_{{\mathrm{f}}2}} \right) = \mathop {\sum}\nolimits_{{\mathrm{ph}}} {{\mathrm{P}}\left( {{\mathrm{ph}}} \right)\mathop {\sum}\nolimits_{{\rm{r}}_{\rm{f}}} {{{P}}\left( {{\mathrm{r}}_{{\mathrm{f}}1},{\mathrm{r}}_{{\mathrm{f}}2}\left| {{\mathrm{ph}}} \right.} \right)} {\mathrm{log}}_2{\textstyle{{{{P}}\left( {{\mathrm{r}}_{{\mathrm{f}}1},{\mathrm{r}}_{{\mathrm{f}}2}\left| {{\mathrm{ph}}} \right.} \right)} \over {{{P}}\left( {{\mathrm{r}}_{{\mathrm{f}}1},{\mathrm{r}}_{{\mathrm{f}}2}} \right)}}}},$$

To assess the degree of independence of the information carried by the two frequencies from the joint information, we computed the information redundancy as follows:

7 $${\mathrm{Red(PH}};{\mathrm{R}}_{{\mathrm{f}}1},{\mathrm{R}}_{{\mathrm{f}}2}{\mathrm{)}} = {I{\mathrm(PH}};{\mathrm{R}}_{{\mathrm{f}}1}{\mathrm{)}} + I{\mathrm{(PH}};{\mathrm{R}}_{{\mathrm{f}}2}{\mathrm{)}} - I{\mathrm{(PH}};{\mathrm{R}}_{{\mathrm{f}}1},{\mathrm{R}}_{{\mathrm{f}}2}{\mathrm{)}}$$

Similar analyses were performed using amplitude/duration of bursts as neural features (see next paragraph for the methods of bursts detection), defined over a given band, rather than the overall power *R*_f_. We corrected for positive information bias as follows^84^: (i) we limited the number of bins of the neural signals to four, to ensure a conservative but stable measure of information; (ii) we applied the Panzeri-Treves bias correction and the shuffling correction in case of joint spectral information; (iii) we compared the resulting values of information with those obtained with 500 bootstrap repetitions, using *p* **<** 0.05 as the information significance threshold. All the aforementioned information measurements were computed using the Matlab^TM^ Information Breakdown Toolbox^84^. To quantify the relevance of common noise source effects, we computed the noise correlation, i.e., the correlation between the trial-by-trial fluctuations around the mean power changes of the reach-to-grasp phases. For both ranges, we computed the trial-by-trial variation, subtracting the average power calculated in each reach-to-grasp phase (i.e., rest, reaching, grasping, and pulling) from the power of each trial. Then we computed the Pearson correlation coefficient between the trial-by-trial variations of the two ranges for each phase and averaged it over all phases. For the sake of completeness, we also computed the percentage change of power in both low and high beta ranges, normalizing the mean power of the reaching phase by subtracting and dividing the mean power of the rest segments in both low and high beta ranges.

### Burst computation and analysis

In line with previous studies^13,30,74,85,86^, we defined as bursts in a given frequency range (or band) the intervals in which the instantaneous amplitude of the range (or band) exceeded the 75th percentile of the signal amplitude distribution across the entire session. Of note, the pattern of results was shown to remain similar regardless of the percentile thresholds used (e.g., 55th to 90th). To check the spectral specificity of our results, we separately computed the wavelet amplitude across the two mutual information-based frequency ranges (i.e., 14 ± 2 Hz and 24 ± 2 Hz), the conventional beta frequency bands (i.e., 13–20 Hz and 21–30 Hz), and two other non-informative bands (theta 4–8 Hz and low gamma 40–60 Hz). Each wavelet amplitude was normalized using a *z*-score operation, then smoothed with a moving average gaussian smoothing kernel of 150 ms. The burst duration was defined as the time spent over the threshold. Bursts shorter than one complete oscillation cycle, e.g., <100 ms for beta bursts, were discarded. The amplitude of a burst was defined as the mean value of the curve above the threshold. Bursts properties during the different reach-to-grasp phases were compared separately for each different range and band. We computed the information quantities described in the previous subsection to determine whether the duration and/or the amplitude of the bursts carried information about the reach-to-grasp phases.

### Statistical analysis

Statistical analyses were performed in Matlab^TM^. The distributions of kinematic variables in PD and healthy controls, did not pass the normality distribution assessment using the Kolmogorov–Smirnov test and were consequently compared with non-parametric test Mann–Whitney *U*-test (ranksum function in Matlab^TM^). For other comparisons within the PD group, we adopted non-parametric Monte Carlo permutation tests (PT). Permutation tests do not rely on assumptions about the underlying data distribution, and the interchanged values always stem from the same physiological source and differ only in the test condition in which they occur. The shuffling procedure was randomly repeated 10,000 times to generate 10,000 mean difference estimates. If the mean difference in the original data was outside the 95% confidence limits of the mean difference of the shuffled data, then this was considered a significant difference. Rank-based Spearman correlations were calculated if data deviated significantly from a normal distribution as assessed by Kolmogorov–Smirnov test. Specifically, we correlated the kinematic measurements of the reaching phase with the striatal DAT density and the beta low-burst and beta high-burst dynamics. Otherwise, linear Pearson correlations were conducted. Statistical analysis on correlation coefficients was conducted after Fisher transformation. The sample size is reported in the main text with the statistical test.

### Reporting summary

Further information on research design is available in the Nature Research Reporting Summary linked to this article.

## Supplementary information

Supplementary Information

Reporting Summary

## Data Availability

Data are available upon reasonable request to the corresponding author.

## Code availability

Custom Matlab^TM^ codes for data analysis are available upon reasonable request to the corresponding author.
