# Supplementary material for: Impaired reach-to-grasp kinematics in parkinsonian patients relates to dopamine-dependent, subthalamic beta bursts
Source: NPJ Parkinsons Dis. 2021 Jun 29;7:53. doi: 10.1038/s41531-021-00187-6 (PMC8242004; doi:10.1038/s41531-021-00187-6)
Supplement: Supplementary file 1 — Supplementary Information [file 41531_2021_187_MOESM1_ESM.docx]

Supplementary Materials

**Supplementary Results**

**Comparison between kinematic features in PD and HC groups**

Subjects with PD showed a similar peak velocity of the wrist in the reaching and pulling phase (0.53±0.07m/s and 0.57±0.06m/s, respectively; n=8; Permutation test [PT, see Methods], p=0.67). The peak velocity in both phases was lower than HC (reaching 0.88±0.06m/s; pulling 0.93±0.05 m/s; n=10; Mann-Whitney U Test, p<0.001 for both; Supplementary Figure 3a). PD patients reached the velocity peak later than HC during both reaching (PD 0.5±0.03s n=8; HC 0.34±0.01s n=10; Mann-Whitney U Test, p<0.001) and pulling phase (PD 0.55±0.03s n=8; HC 0.43±0.03s n=10; Mann-Whitney U Test, p<0.01; Supplementary Figure 3b). HC only displayed a difference in the time to reach the velocity peak in the reaching vs. pulling phase (PT, p<0.01). Differences between HC and PD, however, were not limited to the expected slowness of PD patients but also involved the inter-joint coordination dynamics necessary to follow a planned trajectory. Further comparisons of the movement coordination of PD patients and HC showed significant differences in the integration between the reaching and the grasping phases. First, the peak hand aperture (see Methods) was lower in parkinsonian patients than in HC (PD 35.65±3.87% n=8; HC 45.96±3.3% n=10; Mann-Whitney U Test, p<0.05; Supplementary Figure 3c). Second, the pre-shape coordination index (see Methods) was also lower in PD than in HC (PD 0.67±0.08 n=8; HC 0.79±0.04 n=10; Mann-Whitney U Test, p<0.01; Supplementary Figure 3d).

**Supplementary Figure 1: Velocity profiles of the reach-to-grasp task.**

Absolute velocity profiles of three markers (i.e., the acromion [shoulder], the lateral epicondyle of the humerus [elbow] and the ulnar styloid [wrist]), averaged over one block of ten reach-to-grasp trials for one representative patient with Parkinson’s disease (PD, wue02, **a**) and one healthy control (HC, **b**). The shaded regions around the mean represent the standard errors of the mean. Light and dark gray shaded rectangle bars represent the rest and grasping phases, respectively.


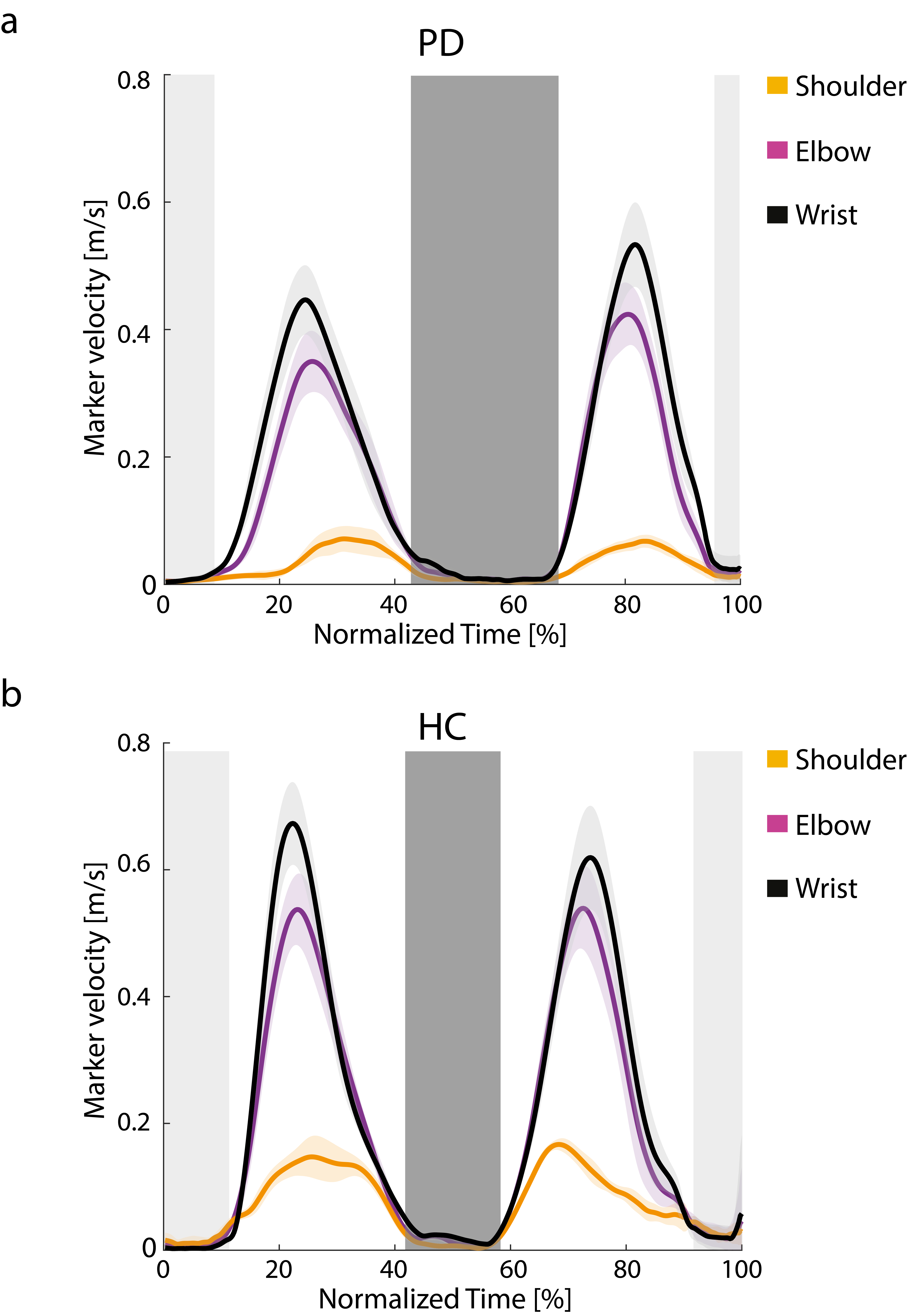


**Supplementary Figure 2: Curvature analysis.**

(**a**) The 3D-trajectory of the ulnar styloid marker (wrist) is displayed for one block (10 trials) of one patient with Parkinson’s disease (PD, wue02). The black arrows indicate the direction of the movement from the start of the reaching phase to the end of the pulling phase. The red points indicate the fractions of movement with the highest value of curvature (>75^th^ percentile) during each trial. (**b**) Comparison between the radius of curvatures (solid lines) and velocity profiles (dashed lines) of one trial during the reaching phase of one PD patient (wue02, black lines) and one healthy control (HC) (gray lines). (**c**) Same as (**b**) for the pulling phase. (**d**) Boxplots of the Fisher-transformed Spearman correlation coefficients between the radius of curvature and the velocity of the ulnar styloid marker over the whole movement for the PD and HC groups. Boxplots are drawn between the 25^th^ and the 75^th^ percentiles, with a horizontal line indicating the median. The whiskers extended above and below to the most extreme data points within 1.5 times the interquartile range. The difference between the two groups was not significant (Mann-Whitney U Test, p>0.05).


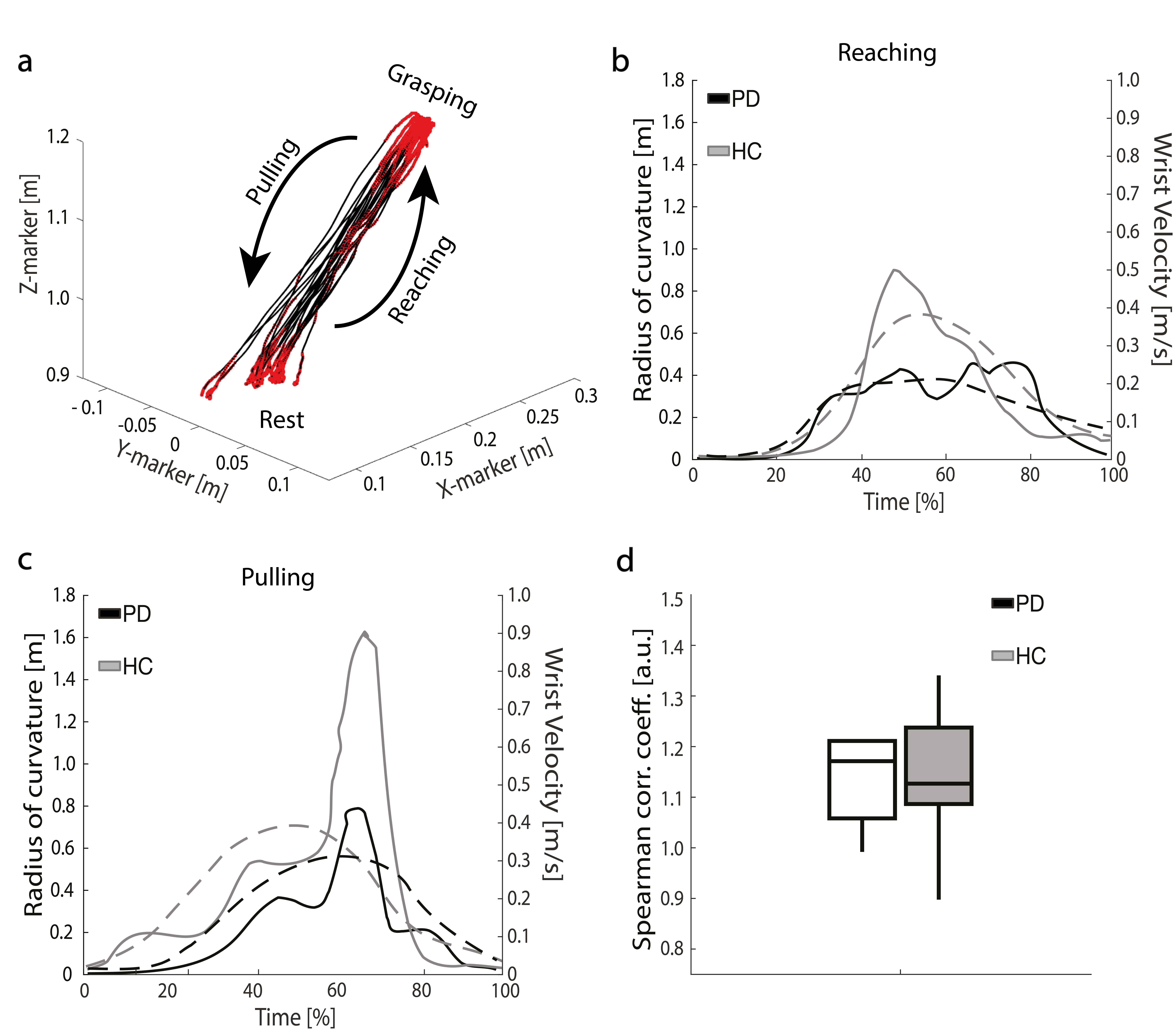


**Supplementary Figure 3: Additional kinematics measurements.**

(**a**) Peak velocity of the wrist marker during the reaching and the pulling phase for the two groups. (**b**) Time to reach peak velocity (wrist marker) during the reaching and the pulling phase. (**c**) Peak hand aperture and (**d**) pre-shape coordination index between Parkinson’s disease group (PD) and Healthy Controls (HC). Boxplots are drawn between the 25th and the 75th percentiles, with a horizontal line indicating the median. The whiskers extended above and below to the most extreme data points within 1.5 times the interquartile range. Asterisks denote the statistical significance *p<0.05, **p<0.01, and ***p<0.001 (PT and Mann-Whitney U Test).

**
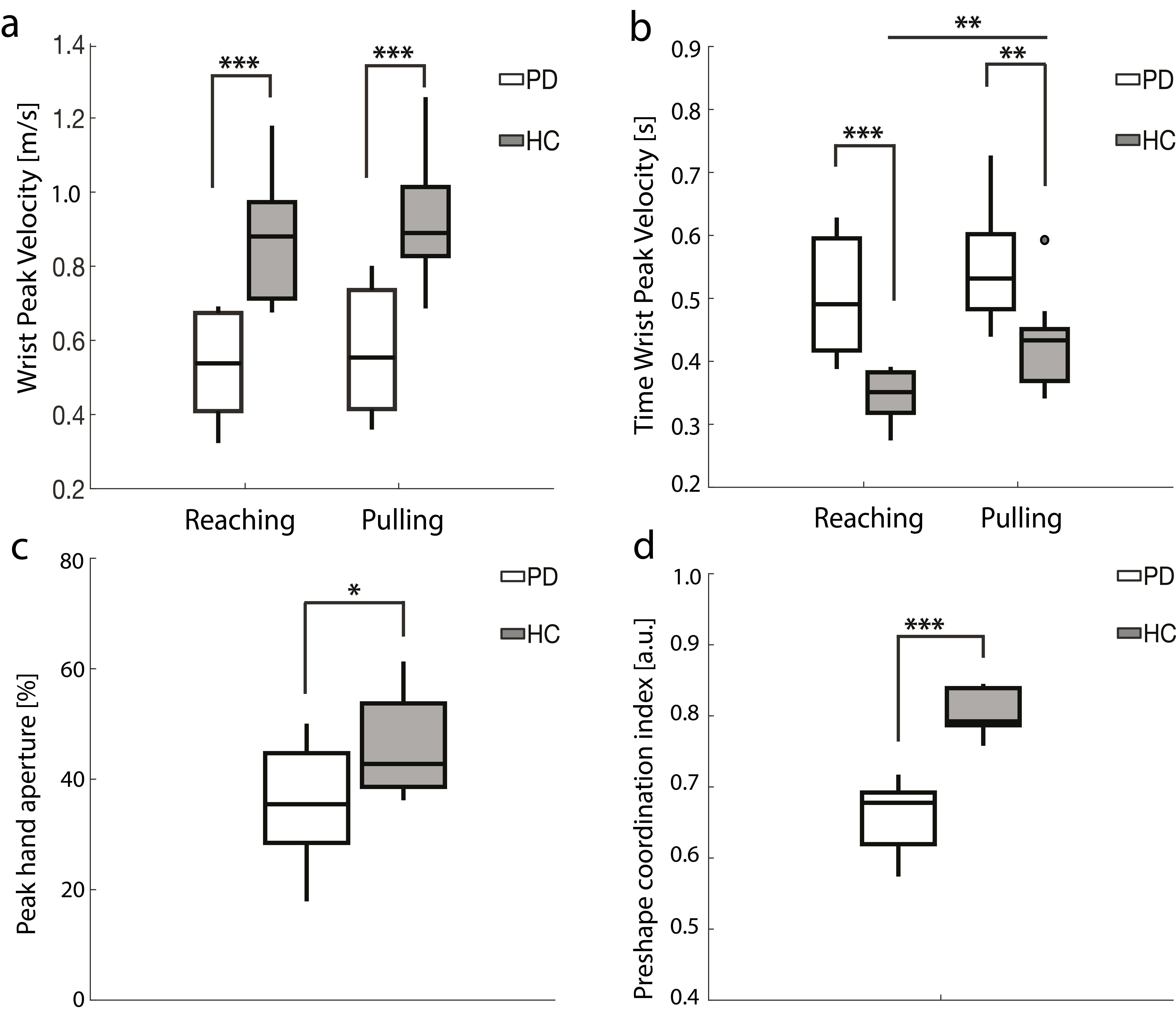
**

**Supplementary Figure 4: Coefficient C-score computation.**

Visual representation of the C-score for one representative trial of patient wue02. Black lines indicate the trajectory during the execution of the task in the angular velocity plan. Black dots indicate lobe centroids. The slope of the dashed black line connecting them is the C-score.


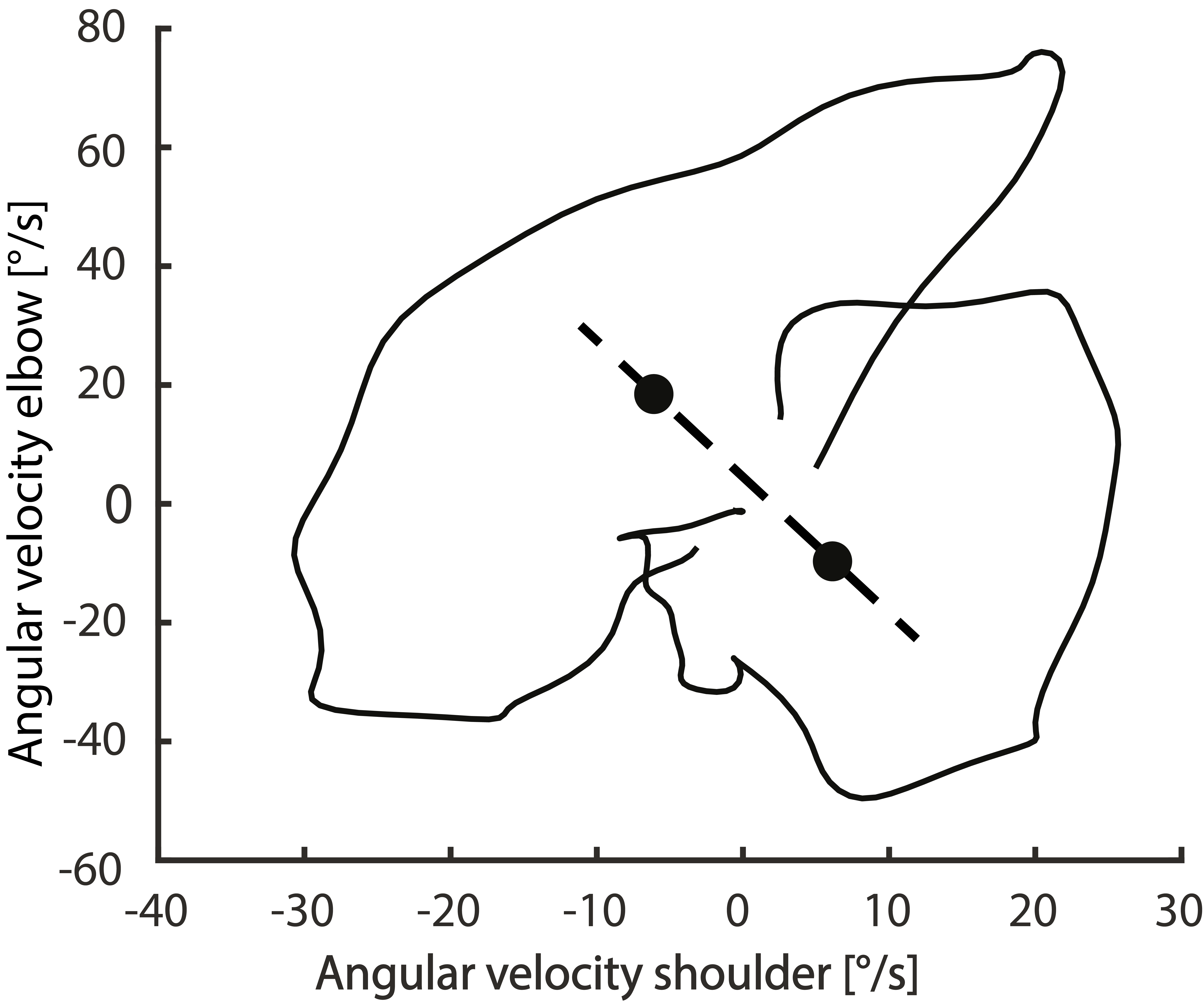


**Supplementary Figure 5: Frequency peak analysis.**

Peak frequencies defined by the power spectral density of each reach-to-grasp phase and maximally informative beta low and beta high frequencies for each patient.


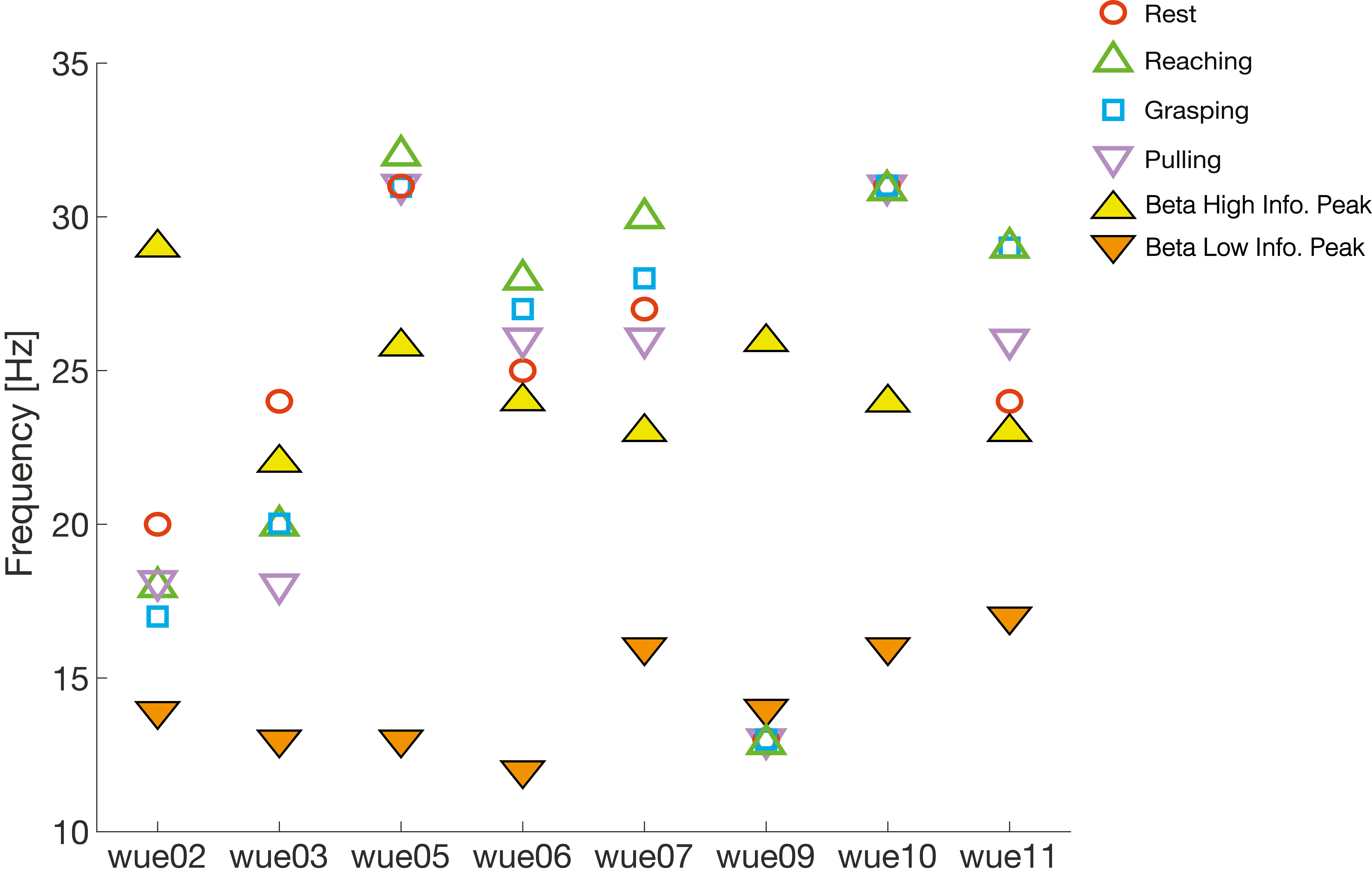


**Supplementary Figure 6: Power across task phases for beta ranges and conventional bands.**

(**a**) Group averaged power in the beta low range (14±2Hz) and in the beta high range (24±2Hz) across the task phases. PT, *p<0.05. (**b**) Group-averaged power in the whole beta band (13–30Hz), in the conventional beta low band (13–20Hz), and in the conventional beta high band (21–30Hz) across the task phases. Boxplots are drawn between the 25^th^ and the 75^th^ percentiles, with a horizontal line indicating the median. The whiskers extended above and below to the most extreme data points within 1.5 times the interquartile range. Asterisks denote the statistical significance *p<0.05, **p<0.01, and ***p<0.001 (PT Test).

**
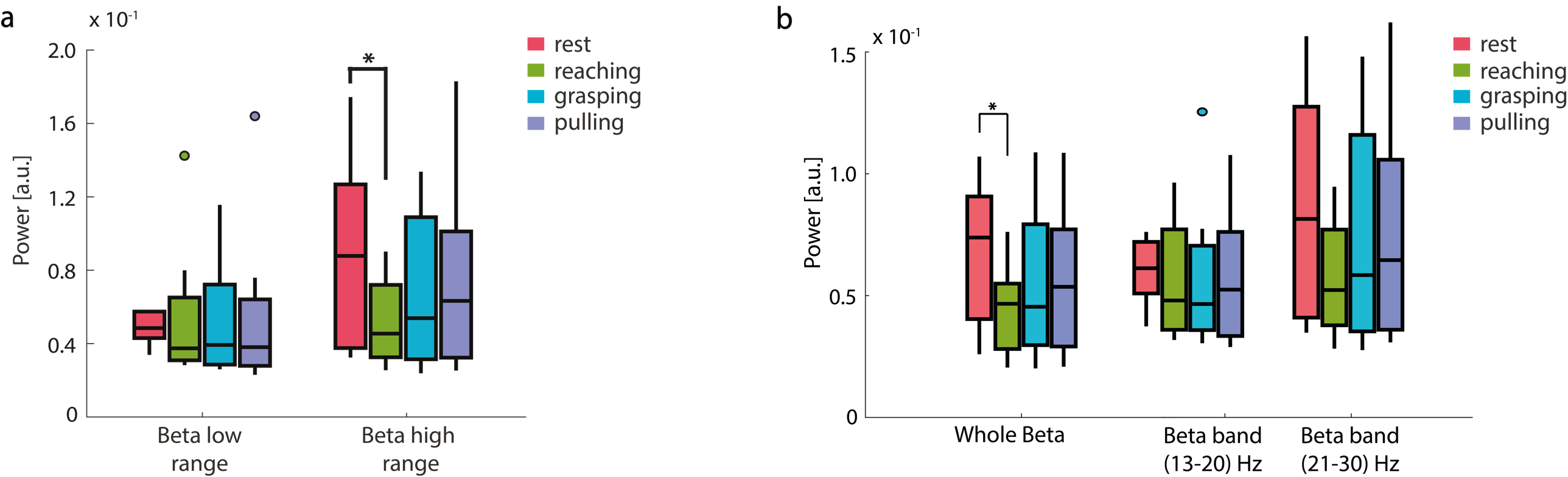
**

**Supplementary Figure 7: Burst analysis in conventional beta bands.**

Same as Figure 3 in the Manuscript but for conventional beta bands: (**a**) Group-averaged burst amplitude in the whole beta band (13–30Hz), in the conventional beta low band (13–20Hz), and in the conventional beta high band (21–30Hz) during the reach-to-grasp task. (**b**) Group-averaged burst duration in the whole beta band (13–30Hz), in the conventional beta low band (13–20Hz), and in the conventional beta high band (21–30Hz) during the reach-to-grasp task. (**c**) Group median information about the reach-to-grasp task carried by the amplitude (gray) and the duration (white) of the beta bursts (conventional bands). Information carried by a shuffle permutation (black) served for statistical significance. Boxplots are drawn between the 25^th^ and the 75^th^ percentiles, with a horizontal line indicating the median. The whiskers extended above and below to the most extreme data points within 1.5 times the interquartile range. Asterisks denote the statistical significance *p<0.05, **p<0.01, and ***p<0.001 (PT Test).

**
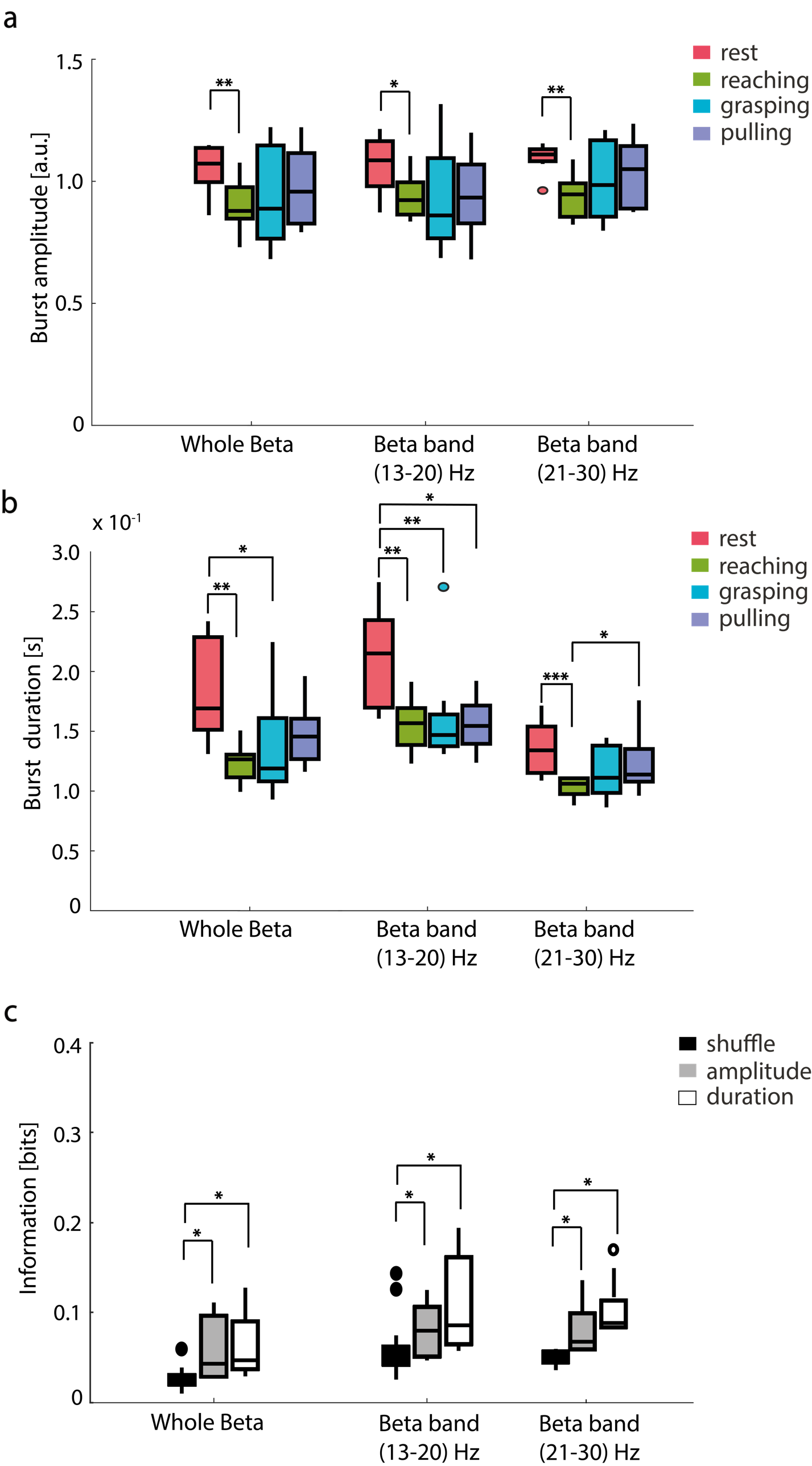
**

**Supplementary Figure 8: Burst analysis for conventional theta and low gamma band.**

(**a**) Group median of power in the conventional theta band (4–8Hz) and low gamma band (30–60Hz) during the reach-to-grasp task. (**b**) Group median of burst amplitude in the conventional theta band (4–8Hz) and low gamma band (30–60Hz) during the reach-to-grasp phases. (**c**) Group median of burst duration in the conventional theta band (4–8Hz) and low gamma band (30–60Hz) during the reach-to-grasp phases. (**d**) Group median information about the reach-to-grasp phases carried by the amplitude (gray) and the duration (white) of the beta bursts. Information carried by a shuffle permutation (black) served for statistical significance. Boxplots are drawn between the 25^th^ and the 75^th^ percentiles, with a horizontal line indicating the median. The whiskers extended above and below to the most extreme data points within 1.5 times the interquartile range. Asterisks denote the statistical significance *p<0.05, **p<0.01, and ***p<0.001 (PT Test).


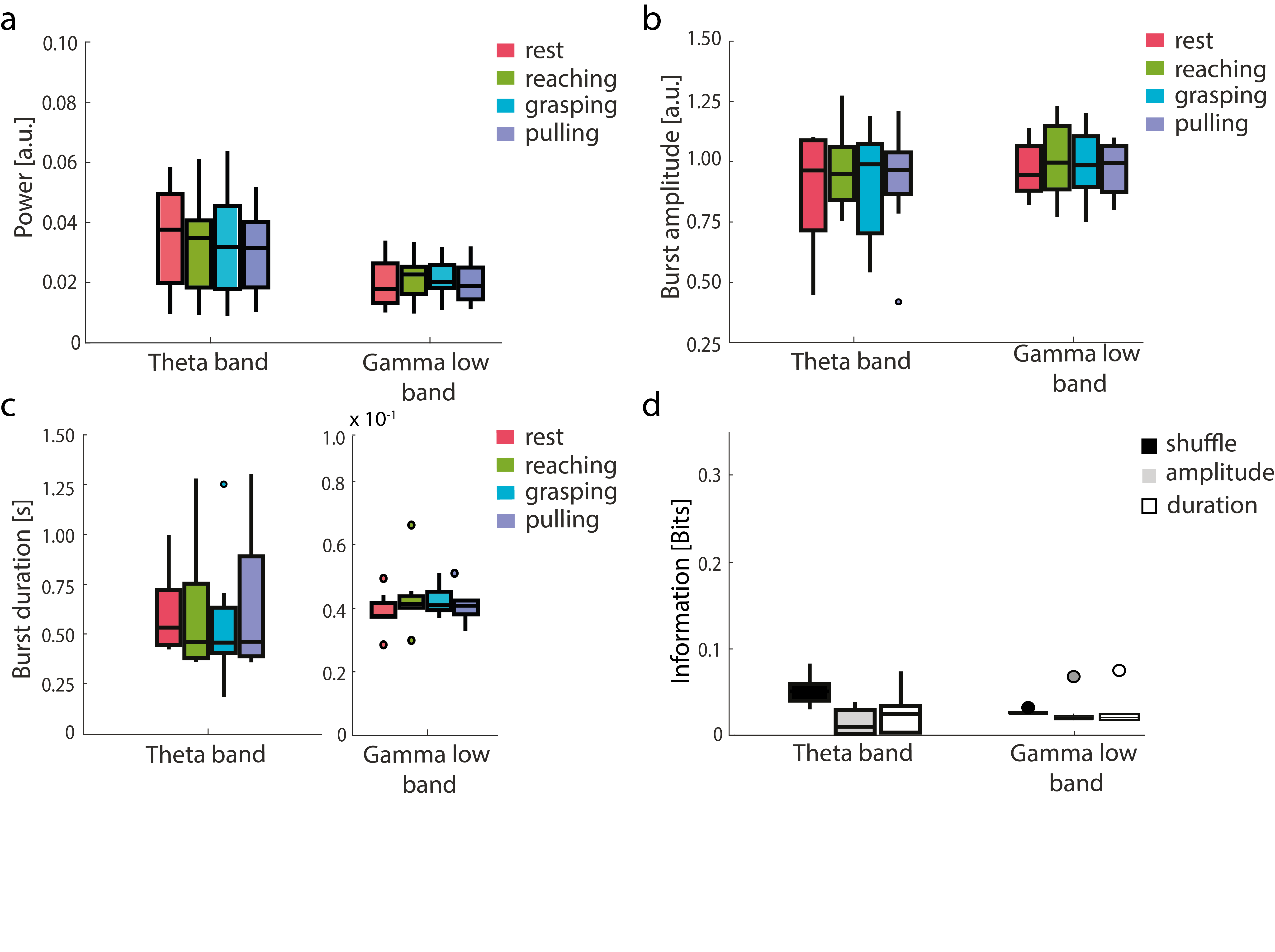


**Supplementary Figure 9: Correlation between C-score and the asymmetry index.**

Pearson correlation between the inter-joint coordination index (C-score) and the inter-hemispheric striatal dopamine loss (asymmetry index). A linear regression (black line) is plotted with 95% confidence intervals (shaded gray regions). Inset reports the correlation value and its statistical significance.

**
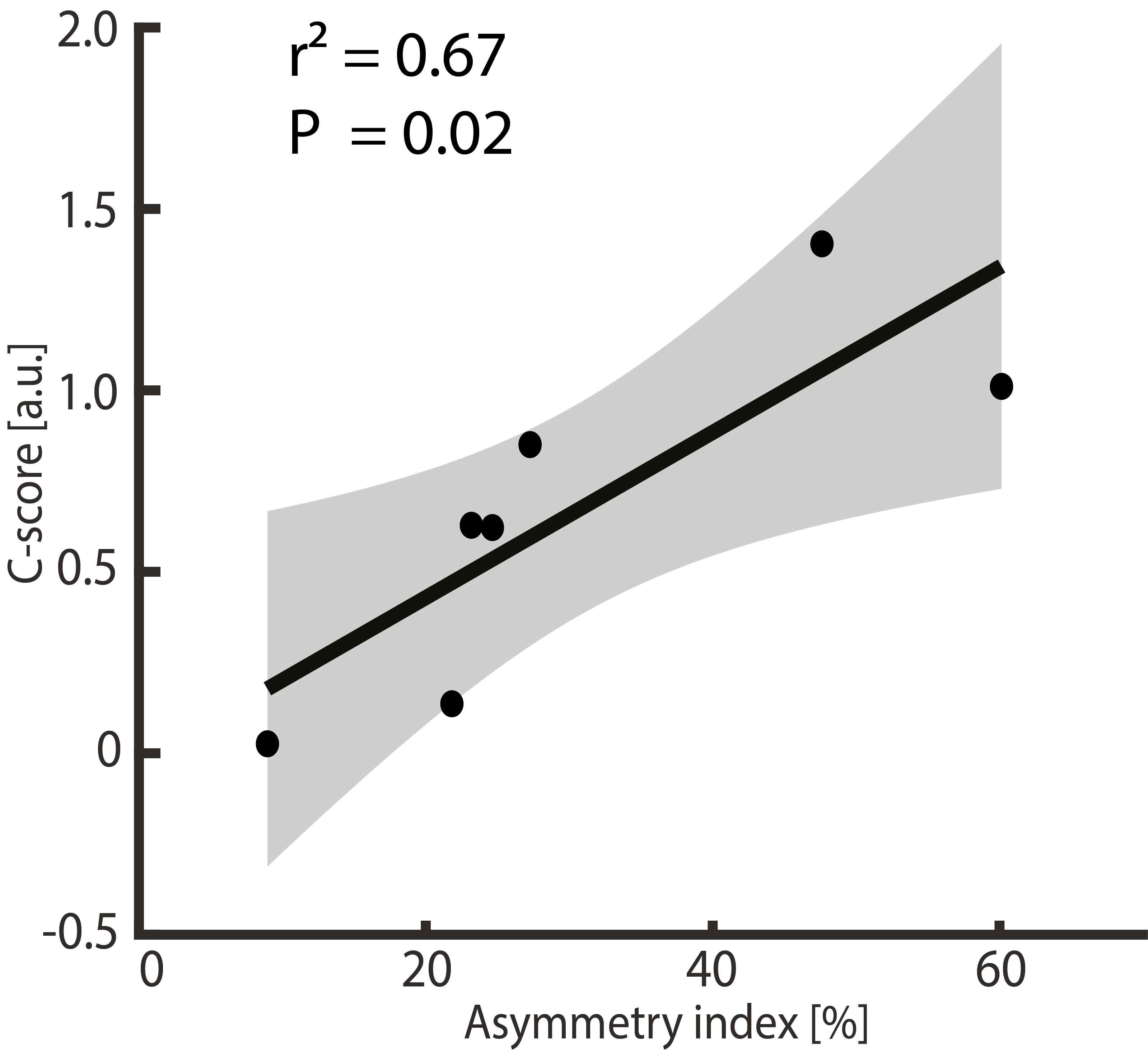
**

**Supplementary Table 1: Demographic and clinical characteristics.**

|  | **Sex** | **Age at surgery (years)** | **Disease duration at surgery (years)** | **LEDD pre-DBS (mg)** | **LEDD post-DBS (mg)** | **UPDRS-III**  **pre-DBS meds-off**  **(score)** | **UPDRS-III**  **pre-DBS meds-on**  **(score)** | **UPDRS-III post-DBS meds-off, stim-off (score)** | **UPDRS-III post-DBS meds-off, stim-on (score)** |
| --- | --- | --- | --- | --- | --- | --- | --- | --- | --- |
| **wue02** | M | 65 | 10 | 1100 | 800 | 40 | 23 | 39 | 19 |
| **wue03** | M | 61 | 18 | 2725 | 600 | 40 | 9 | 45 | 17 |
| **wue05** | M | 67 | 17 | 1050 | 500 | 49 | 24 | 47 | 13 |
| **wue06** | M | 51 | 11 | 1133 | 180 | 46 | 11 | 48 | 12 |
| **wue07** | M | 61 | 10 | 650 | 220 | 43 | 24 | 29 | 15 |
| **wue09** | M | 55 | 19 | 1200 | 730 | 50 | 11 | 33 | 16 |
| **wue10** | M | 56 | 10 | 1200 | 550 | 69 | 14 | 65 | 25 |
| **wue11** | F | 53 | 11 | 1300 | 460 | 55 | 4 | 51 | 9 |
| **Mean (SD)** |  | 58.62  (5.43) | 13.25  (3.72) | 1294.75  (570.45) | 505  (219.61) | 49.12  (8.94) | 15.00  (7.21) | 44.63  (10.51) | 15.75  (4.54) |

Before surgery, all patients were evaluated using the UPDRS-III scale after overnight withdrawal (12h) of all dopaminergic medications (pre-DBS, meds-off) and about 1h after the intake of 1–1.5 times the levodopa-equivalent of the preoperative morning dose (pre-DBS, meds-on). After surgery, all patients were evaluated in the meds-off condition with chronically effective stimulation (post-DBS meds-off, stim-on) and after pausing the stimulation for 2h (post-DBS meds-off, stim-off). DBS, deep brain stimulation; LEDD, levodopa equivalent daily dose; SD, standard deviation; STN, subthalamic nucleus; UPDRS-III, Unified Parkinson Disease Rating Scale part III (motor part).

**Supplementary Table 2: Molecular imaging findings.**

|  | **Left hemisphere** | | | **Right hemisphere** | | | **Asymmetry index** |
| --- | --- | --- | --- | --- | --- | --- | --- |
|  | **Caudate n.** | **Putamen** | **Striatum** | **Caudate n.** | **Putamen** | **Striatum** | **Striatum** |
| **wue02** | 0.88 | 0.57 | 0.72 | 1.43 | 0.91 | 1.17 | 47.62 |
| **wue03** | 1.15 | 0.70 | 0.93 | 0.58 | 0.44 | 0.50 | 60.14 |
| **wue05** | - | - | - | - | - | - | - |
| **wue06** | 1.31 | 0.59 | 0.95 | 1.56 | 0.91 | 1.20 | 23.26 |
| **wue07** | 0.92 | 0.64 | 0.76 | 1.22 | 0.79 | 1.00 | 27.27 |
| **wue09** | 0.72 | 0.48 | 0.61 | 0.62 | 0.37 | 0.49 | 21.82 |
| **wue10** | 0.97 | 0.50 | 0.75 | 1.20 | 0.74 | 0.96 | 24.56 |
| **wue11** | 1.15 | 0.79 | 0.96 | 1.41 | 0.74 | 1.05 | 8.96 |
| **PD mean (SD)** | 1.01 (0.19) | 0.61 (0.10) | 0.81 (0.13) | 1.15 (0.36) | 0.70 (0.19) | 0.91 (0.27) | 30.52 (16.07) |
| **Healthy reference mean (SD)** | 2.61 (0.52) | 2.23 (0.48) | 2.30 (0.48) | 2.57 (0.57) | 2.29 (0.4) | 2.33 (0.48) | 2.57 (2.14) |

Non-displaceable binding potentials of dopamine reuptake transporters (DAT) values for left and right striatum, caudate nucleus, and putamen. Wue05 did not perform the molecular imaging study. As references, we used the molecular imaging values of a group of healthy subjects (4 males, 11 females; age range 44–68 years). SD, standard deviation.

**Supplementary Table 3: Most informative beta low and beta high frequency for each patient.**

|  | **Beta low (Hz)** | **Beta high (Hz)** |
| --- | --- | --- |
| **Wue02** | 14 | 29 |
| **Wue03** | 13 | 22 |
| **Wue05** | 13 | 26 |
| **Wue06** | 12 | 24 |
| **Wue07** | 16 | 23 |
| **Wue09** | 14 | 26 |
| **Wue10** | 16 | 24 |
| **Wue11** | 17 | 23 |
| **Mean ± SD** | 14.38 ± 1.78 | 24.62 ± 2.26 |

Data are reported as mean ± standard deviation (SD).

**Supplementary Table 4a: Correlation between spatiotemporal kinematics of the reach-to-grasp task and beta high-range bursts features.**

|  | **Wrist peak velocity** | **Time to wrist peak velocity** | **Radius of curvature** |
| --- | --- | --- | --- |
| **Beta high-range burst amplitude** | R^2^ =0.32, p=0.14 | R^2^ =0.42, p=0.08 | R^2^ =0.84, p=0.006 |
| **Beta high-range burst duration** | R^2^ =0.02, p>0.50 | R^2^ =0.25, p=0.22 | R^2^ =0.21, p=0.25 |

Results are shown as R-squared and p-value FDR-corrected of the correlation analysis.

**Supplementary Table 4b: Correlation between coordinative aspects of the reach-to-grasp task and beta high-range bursts features.**

|  | **Peak hand aperture** | **Pre-shape coordination index** | **C-score** |
| --- | --- | --- | --- |
| **Beta high-range burst amplitude** | R^2^=0.28, p=0.17 | R^2^=0.02, p>0.50 | R^2^=0.02, p>0.50 |
| **Beta high-range burst duration** | R^2^=0.02, p>0.50 | R^2^=0.01, p=0.50 | R^2^=0.01, p>0.50 |

Results are shown as R-squared and p-value FDR-corrected of the correlation analysis. C-score = inter-joint coordination index.

**Supplementary Table 5: Correlation between spatiotemporal kinematics of the reach-to-grasp task and beta high-range modulations.**

|  | **AI** | **C-score** |
| --- | --- | --- |
| **Beta high-range power modulation (w.r.t. rest)** | R^2^ =0.50, p=0.07 | R^2^ =0.47, p=0.08 |
| **Beta high-range burst amplitude modulation (w.r.t. rest)** | R^2^ =0.36, p=0.16 | R^2^ =0.23, p=0.22 |
| **Beta high-range burst duration modulation (w.r.t. rest)** | R^2^ =0.24, p=0.26 | R^2^ =0.22, p=0.25 |
| **Beta high-range burst (amplitude+duration) modulation (w.r.t. rest)** | R^2^ =0.32, p=0.18 | R^2^ =0.24, p=0.22 |

Results are shown as R-squared and p-value FDR-corrected of the correlation analysis. AI = Asymmetry Index; C-score = inter-joint coordination index; w.r.t=with respect to rest.

**Supplementary Table 6a: Correlation between striatal DAT density and spatiotemporal kinematics of the reach-to-grasp task.**

|  | **Wrist peak velocity** | **Time to wrist peak velocity** | **Radius of curvature** |
| --- | --- | --- | --- |
| **Striatal DAT density (left hemisphere)** | R^2^=0.36, p=0.11 | R^2^=0.73, p=0.01 | R^2^=0.03, p>0.50 |
| **Striatal DAT density (hemispheres average)** | R^2^=0.05, p>0.50 | R^2^=0.05, p>0.50 | R^2^=0.09, p>0.50 |

Results are shown as R-squared and p-value FDR-corrected of the correlation analysis. DAT = dopamine reuptake transporter.

**Supplementary Table 6b: Correlation between striatal DAT density and coordinative kinematics of the reach-to-grasp task.**

|  | **Peak hand aperture** | **Pre-shape coordination index** | **C-score** |
| --- | --- | --- | --- |
| **Striatal DAT density (left hemisphere)** | R^2^=0.01, p>0.5 | R^2^=0.05, p>0.5 | R^2^=0.07, p>0.5 |
| **Striatal DAT density (hemispheres average)** | R^2^=0.11, p>0.5 | R^2^=0.07, p>0.5 | R^2^=0.12, p>0.5 |

Results are shown as R-squared and p-value FDR-corrected of the correlation analysis. DAT = dopamine reuptake transporter.

**Supplementary Table 7: Correlation between striatal density and beta high- and low-range bursts features.**

|  | **Striatal DAT density (left hemisphere)** | **Striatal DAT density**  **(hemispheres average)** |
| --- | --- | --- |
| **Beta low-range burst amplitude** | R^2^ =0.02, p>0.50 | R^2^ =0.18, p=0.35 |
| **Beta low-range burst duration** | R^2^ =0.43, p=0.11 | R^2^ =0.28, p=0.22 |
| **Beta high-range burst amplitude** | R^2^ =0.01, p>0.50 | R^2^ =0.01, p>0.50 |
| **Beta high-range burst duration** | R^2^ =0.02, p>0.50 | R^2^ =0.02, p=0.22 |

Results are shown as R-squared and p-value FDR-corrected of the correlation analysis. DAT = dopamine reuptake transporter.
