# Supplementary material for: Impaired reach-to-grasp kinematics in parkinsonian patients relates to dopamine-dependent, subthalamic beta bursts
Source: NPJ Parkinsons Dis. 2021 Jun 29;7:53. doi: 10.1038/s41531-021-00187-6 (PMC8242004; doi:10.1038/s41531-021-00187-6)
Supplement: Supplementary file 2 — Reporting Summary [file 41531_2021_187_MOESM2_ESM.pdf]

nature research

Corresponding author(s): Ioannis Ugo ISAIAS

Last updated by author(s): Mar 3, 2021

Reporting Summary

Nature Research wishes to improve the reproducibility of the work that we publish. This form provides structure for consistency and transparency in reporting. For further information on Nature Research policies, see our Editorial Policies and the Editorial Policy Checklist.

Statistics

For all statistical analyses, confirm that the following items are present in the figure legend, table legend, main text, or Methods section.

n/a	Confirmed	
☐	☒	The exact sample size (*n*) for each experimental group/condition, given as a discrete number and unit of measurement
☐	☒	A statement on whether measurements were taken from distinct samples or whether the same sample was measured repeatedly
☐	☒	The statistical test(s) used AND whether they are one- or two-sided<br>*Only common tests should be described solely by name; describe more complex techniques in the Methods section.*
☐	☒	A description of all covariates tested
☐	☒	A description of any assumptions or corrections, such as tests of normality and adjustment for multiple comparisons
☐	☒	A full description of the statistical parameters including central tendency (e.g. means) or other basic estimates (e.g. regression coefficient) AND variation (e.g. standard deviation) or associated estimates of uncertainty (e.g. confidence intervals)
☐	☒	For null hypothesis testing, the test statistic (e.g. F, t, r) with confidence intervals, effect sizes, degrees of freedom and P value noted<br>*Give P values as exact values whenever suitable.*
☒	☐	For Bayesian analysis, information on the choice of priors and Markov chain Monte Carlo settings
☒	☐	For hierarchical and complex designs, identification of the appropriate level for tests and full reporting of outcomes
☐	☒	Estimates of effect sizes (e.g. Cohen's d, Pearson's r), indicating how they were calculated

Our web collection on statistics for biologists contains articles on many of the points above.

Software and code

Policy information about availability of computer code

Data collection	SMART-DX BTS, SureTuneTM Medtronic PLC, Activa PC+S® system Medtronic PLC
Data analysis	Data analysis was performed in Matlab environment. Custom scripts are available on request.

For manuscripts utilizing custom algorithms or software that are central to the research but not yet described in published literature, software must be made available to editors and reviewers. We strongly encourage code deposition in a community repository (e.g. GitHub). See the Nature Research guidelines for submitting code & software for further information.

Data

Policy information about availability of data

All manuscripts must include a data availability statement. This statement should provide the following information, where applicable:

- Accession codes, unique identifiers, or web links for publicly available datasets
- A list of figures that have associated raw data
- A description of any restrictions on data availability

Data are available upon reasonable request to the corresponding author.

April 2020

Field-specific reporting

Please select the one below that is the best fit for your research. If you are not sure, read the appropriate sections before making your selection.

☒ Life sciences ☐ Behavioural & social sciences ☐ Ecological, evolutionary & environmental sciences

For a reference copy of the document with all sections, see nature.com/documents/nr-reporting-summary-flat.pdf

Life sciences study design

All studies must disclose on these points even when the disclosure is negative.

Sample size	We recruited all parkinsonian patients implanted at the University Hospital Würzburg with the Activa PC+S® device. This was a prototype available only in very few number for therapeutic DBS as well as on-demand LFP recordings from the implanted electrodes.
Data exclusions	No data were excluded.
Replication	Replication was not possible with our dataset. We provided a detailed description of all methods thus allowing other Centers, where the Activa PC+S® device is available, to replicate our findings.
Randomization	Randomizaton does not apply.
Blinding	Blinding was not relevant.

Reporting for specific materials, systems and methods

We require information from authors about some types of materials, experimental systems and methods used in many studies. Here, indicate whether each material, system or method listed is relevant to your study. If you are not sure if a list item applies to your research, read the appropriate section before selecting a response.

Materials & experimental systems

n/a	Involved in the study	
☒	☐	Antibodies
☒	☐	Eukaryotic cell lines
☒	☐	Palaeontology and archaeology
☒	☐	Animals and other organisms
☐	☒	Human research participants
☐	☒	Clinical data
☒	☐	Dual use research of concern

Methods

n/a	Involved in the study	
☒	☐	ChIP-seq
☒	☐	Flow cytometry
☒	☐	MRI-based neuroimaging

Human research participants

Policy information about studies involving human research participants

Population characteristics	We recruited patients suffering from idiopathic Parkinson's disease. We did not identify any demographic covariate relevant for this study. With regards to clinical features (e.g., bradykinesia and rigidity), we used their brain imaging or kinematic counterpart being more precise assessments.
Recruitment	We consecutively recruited parkinsonian patients in need (clinical indication) of STN DBS.
Ethics oversight	University Hospital Würzburg

Note that full information on the approval of the study protocol must also be provided in the manuscript.

Clinical data

Policy information about clinical studies

All manuscripts should comply with the ICMJE guidelines for publication of clinical research and a completed CONSORT checklist must be included with all submissions.

Clinical trial registration	Not applicable. Our study was not a clinical trial.
Study protocol	The full trial protocol is described in the text.
Data collection	Recording were performed at the University Hospital Würzburg between 2015 and 2018.

Outcomes	Outcomes identification and selection are fully described in the text. In particular, kinematic, LFP and molecular imaging measurements were predefined based on our previous experience and literature data. Information theory analysis was also applied following our previous experience with these metrics.
